# Modeling the activation of the alternative complement pathway and its effects on hemolysis in health and disease

**DOI:** 10.1371/journal.pcbi.1008139

**Published:** 2020-10-02

**Authors:** Antonello Caruso, Jannik Vollmer, Matthias Machacek, Elod Kortvely

**Affiliations:** 1 Roche Pharma Research and Early Development, Pharmaceutical Sciences, Roche Innovation Center Basel, F. Hoffmann-La Roche Ltd, Basel, Switzerland; 2 LYO-X GmbH, Allschwil, Switzerland; 3 Roche Pharma Research and Early Development, Immunology, Infectious Diseases and Ophthalmology (I2O) Discovery and Translational Area, Roche Innovation Center Basel, F. Hoffmann-La Roche Ltd, Basel, Switzerland; University of Pittsburgh, UNITED STATES

## Abstract

The complement system is a powerful mechanism of innate immunity poised to eliminate foreign cells and pathogens. It is an intricate network of >35 proteins, which, once activated, leads to the tagging of the surface to be eliminated, produces potent chemoattractants to recruit immune cells, and inserts cytotoxic pores into nearby lipid surfaces. Although it can be triggered via different pathways, its net output is largely based on the direct or indirect activation of the alternative pathway. Complement dysregulation or deficiencies may cause severe pathologies, such as paroxysmal nocturnal hemoglobinuria (PNH), where a lack of complement control proteins leads to hemolysis and life-threatening anemia. The complexity of the system poses a challenge for the interpretation of experimental data and the design of effective pharmacological therapies. To address this issue, we developed a mathematical model of the alternative complement pathway building on previous modelling efforts. The model links complement activation to the hemolytic activity of the terminal alternative pathway, providing an accurate description of pathway activity as observed in vitro and in vivo, in health and disease. Through adjustment of the parameters describing experimental conditions, the model was capable of reproducing the results of an array of standard assays used in complement research. To demonstrate its clinical applicability, we compared model predictions with clinical observations of the recovery of hematological biomarkers in PNH patients treated with the complement inhibiting anti-C5 antibody eculizumab. In conclusion, the model can enhance the understanding of complement biology and its role in disease pathogenesis, help identifying promising targets for pharmacological intervention, and predict the outcome of complement-targeting pharmacological interventions.

## Author summary

The complement system as a part of the innate immunity builds a first line of defense of the body against foreign and altered host structures, such as pathogens and damaged cells. In contrast to the adaptive immune system, which takes days to weeks to mount a response, the complement system can react within seconds to minutes and therefore enables the body to quickly react to infections. Dysregulation of the system, however, has been identified as a major driver or significant contributor to several pathologies, ranging from the pathologic lysis of body’s own erythrocytes to the progressive loss of eyesight. The pathway’s highly diverse and interconnected architecture makes it difficult to identify promising points of therapeutic intervention and to predict clinical study outcomes. Building on previous modelling efforts, in this work we developed a mathematical model of the alternative pathway of the complement system that can explain experimental observations acquired over more than 50 years of complement research. These include results acquired in the laboratory as well as in patients. We envision that this work can be used to support the development of novel treatments for complement-mediated diseases at several key points such as target selection or design of clinical studies.

## Introduction

As a first responder of innate immunity, the complement system, a network of more than 35 proteins, provides a powerful mechanism to mark and eliminate unwanted foreign and self-structures [1]. Complement activation triggers a proteolytic cascade leading to the production of cleavage products with various biological functions [2,3]. Diffusible fragments (such as the anaphylatoxins) trigger degranulation of neighboring endothelial and mast cells, increase the permeability of capillaries and recruit immune cells (chemotaxis) [4]. Furthermore, complement actively tags surfaces destined for elimination by covalent attachment of specific fragments to the activating surface [5]. Finally, it is actively involved in the disruption of pathogens owing to the insertion of pore-forming complement proteins into the membrane of the invading microorganism [6].

Three pathways may activate the complement system, depending on the mechanism triggering response. Antigen-bound antibodies and sugar moieties initiate the classical (CP) and lectin pathway (LP), respectively [7]. The third pathway, the alternative pathway (AP), does not require a specific activator. Instead, it is always active at low level (tick-over) [8–11] and continuously challenges any surface it encounters, including self-cells. Healthy cells can counterbalance this pressure, while damaged cells or pathogens are rapidly eliminated. All three pathways lead to the cleavage of complement component 3 (C3) into C3a and C3b, with the difference that the composition of the enzyme complex catalyzing this reaction is different for the CP/LP and the AP. The generated C3b becomes a part of the AP C3 convertase (C3bBb), responsible for cleaving C3 and thereby providing amplification for the activation of the other pathways. Amplification by AP may actually account for up to 80% of complement effector functions, irrespective of the pathway initially activated [12].

The complement system can eliminate cells within minutes, while the adaptive immune system requires weeks to mount an immune response. Complement can however harm self-structures when its regulation is out of balance, making it a major driver in several pathologies [13]. The rare kidney disease paroxysmal nocturnal hemoglobinuria (PNH) is a prominent example of a complement-driven disease [14]. In PNH, the lack of negative complement regulator proteins at the surface of erythrocytes leaves them unprotected against complement attack [15–17], leading to continual hemolysis and life-threatening anemia. The US Food and Drug Administration (FDA) approved eculizumab (Soliris), a complement-targeting monoclonal antibody for treatment of PNH and later for a similarly rare kidney disease, atypical hemolytic uremic syndrome (aHUS). This success led to a deluge of clinical trials targeting the complement system [18–20]. The genetic architecture of age-related macular degeneration (AMD), the most prevalent blinding disease of the elderly, also suggests that complement plays a pivotal role in the pathogenesis [21]. Protein-changing polymorphisms in several complement genes were found to increase the risk of developing this sight-threatening condition [22–27], albeit the molecular underpinnings of the manifestation of the disease are poorly understood. Strikingly, a coding SNP in the complement protein Factor H (FH) is one of the two major susceptibility loci, and the encoded negative regulator is responsible for holding the AP at bay [28–32]. Besides, polymorphisms affecting the activity of Factor B (FB), a serine protease exclusive to the AP, were found to have a direct impact on disease risk further supporting the pronounced role of this pathway in the etiology of AMD [33].

Nevertheless, due to the cascade nature of the complement system and the numerous proteins, activation products, positive and negative regulators, as well as transient protein complexes involved, finding promising points of intervention and predicting the outcome of clinical trials remains a challenging task [34]. A drug candidate may excel when its efficacy is assessed in an ex vivo assay (e.g. in a blood sample) and fall short in vivo where the constant synthesis and elimination (homeostasis) of complement proteins also influence the outcome, among other factors [34]. This high degree of complexity prevents simple interpretations and renders intuitive predictions of complement activation and regulation unreliable. On the other hand, the growing number of drugs in development pipelines necessitates the organization of all relevant components into a testable framework.

Mathematical models are often employed to facilitate the study of complex systems. Modeling techniques offer a means to formalize the available biological knowledge into a quantitative description of the system dynamics and obtain a reproducible testbed. A few models of the complement system have been developed, aiming to enhance our understanding of homeostasis and regulation [35–41]. Hirayama and co-workers [35] first studied CP activation by linear systems analysis to investigate its stability and controllability. Korotaevskiy and colleagues [36] investigated the bactericidal dynamics of the CP and AP and Liu and colleagues [37] that of the CP and LP. More recently, Sagar and colleagues [38] developed a reduced model for the LP and AP capable of mimicking C3a and C5a kinetics in vitro, but which did not describe the terminal pathway beyond the formation of the C5 convertase. Bakshi and co-workers [41] developed two parsimonious models of the AP that took into account the synthesis and degradation of precursors with the aim to understand the steady-state response of the pathway. A model of the AP and CP that aimed at including all relevant molecules and reactions was presented by Zewde and colleagues [39,40], describing pathway interactions with pathogen surfaces and host cells in an in vitro setting. However, none of the published models studied the role of the AP as a driver of disease while aiming to reproduce experimental observations obtained in vitro and in vivo.

The aim of this work was to develop a mathematical model of the AP that allows to investigate complement activation as measured in vitro in laboratory assays and in vivo via clinical markers of disease. The goal was to develop a model that contains all relevant molecules and reactions as opposed to a reductionist approach. Such a model would allow probing the pathway at different nodes and facilitate the comparison to current and emerging experimental data. Being the most comprehensive model of the AP to date, the work by Zewde and colleagues [39] was used as a basis for this work. This mathematical model was evolved by estimating model parameters to fit an extensive set of experimental observations and by incorporating several disease relevant readouts, such as hemolysis of rabbit and human erythrocytes and hematological biomarkers (hemoglobin, LDH and hematocrit). By doing so, it allowed to compare the model predictions to the results of commonly used experimental setups and in vivo observations. The focus was the AP because of its prominent contribution to the generation of complement activation products, as well as to balance biological completeness and mathematical tractability. An ordinary differential equation (ODE) model was formulated and its predictive accuracy assessed under a range of experimental conditions, representing both the in vitro and in vivo, healthy and diseased setting using all published experimental data that was identified from peer-reviewed publications.

Hemolytic assays quantify the functional activity of the complement system by measuring the degree of erythrocyte lysis upon incubation in serum. They have been the cornerstone of complement research for over 50 years [42]. Therefore, we included complement-mediated hemolysis into the model and assessed the predictions against experimental measurements. The turnover of complement proteins and erythrocytes was also taken into account to mimic the metabolic processes that underpin homeostasis in vivo. As a result, the model can simulate the pathological changes induced by complement dysregulation in conditions like PNH, and how these are reflected in clinical markers of disease, such as hemoglobin concentration and hematocrit levels. With the addition of a mathematical description of eculizumab pharmacokinetics and target engagement, we finally simulated the pharmacological inhibition of complement-mediated hemolysis and compared it to clinical observations of hemoglobin recovery in PNH patients.

In summary, the AP model provides a platform to investigate pathway activity in vitro and in vivo and predict the outcomes of pathway modulation by disease and pharmacological intervention. The model may be useful to identify promising targets for drug development and assist the selection of biomarkers of clinical efficacy. It may also guide the definition of personalized treatments in the future, assuming the model can be adapted to match data from individual patients, and be used to study the optimal administration of drugs.

## Methods

### Alternative pathway model

#### Model structure

**Fig 1:**
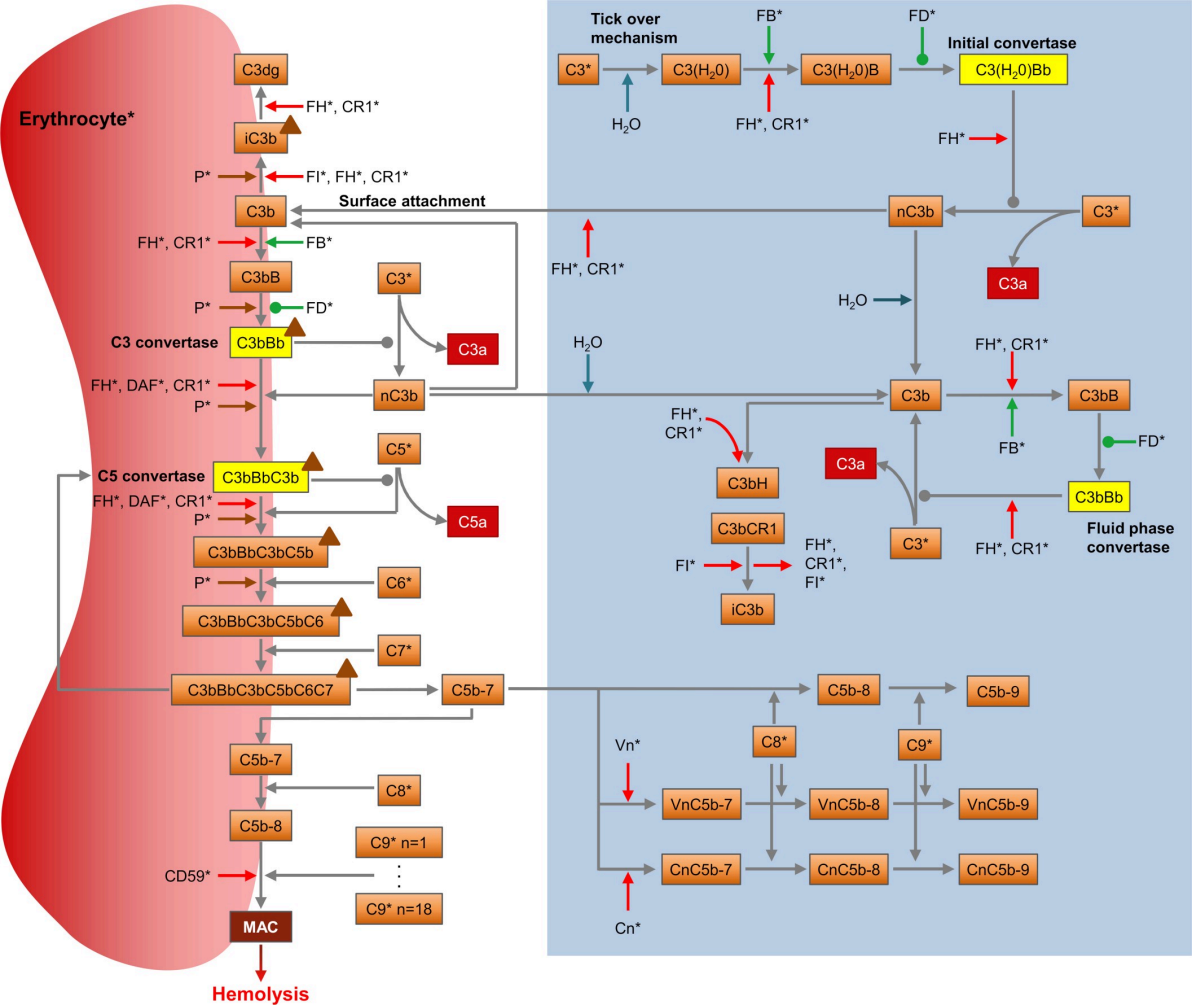
The alternative pathway of the complement system. The diagram shows the pathway as described in the model with its components (orange boxes), reactions (grey arrows), proactivators (green arrows), regulators (red arrows), convertases (yellow boxes), effectors (red boxes), and the positive regulator properdin (brown triangles). Association/dissociation reactions are displayed with a pointed arrowhead, enzymatic reactions with an oval. Degradation was implemented for all proteins and complexes. Proteins marked with a (*) are synthesized. The blue box indicates the reactions of the fluid phase in absence of erythrocytes. Activation of the AP begins with spontaneous hydrolysis (“tick-over”) of C3 producing C3(H_2_O), which in turn can form the initial convertase C3(H_2_O)Bb in presence of FB and factor D (FD). The initial convertase can generate more C3b through C3 cleavage. C3b may bind FB, leading to the formation of the fluid-phase convertase C3bBb, or attach to a nearby surface, such as a cell membrane. Surface-attached C3b can combine with FB to form the surface C3 convertase C3bBb, reacting with C3b to generate the C5 convertase, C3bBbC3b. Cleavage of C5 by the C5 convertase activates the terminal pathway. The anaphylatoxin C5a is released while C5b remains attached to the C5 convertase, followed by C6 and C7 binding. The complex C5b-7 is released into the fluid phase, from where it can reinsert into the cell membrane if not sequestered by the regulators vitronectin (Vn) or clusterin (Cn). Upon binding of membrane C5b-7 to C8 and up to 18 C9 molecules [42], the membrane attack complex (MAC) is formed. With sufficient terminal pathway activity, the cell is lysed as a result of the accumulation of MAC complexes, permitting free diffusion of molecules across its membrane. All negative regulators (FH, decay-accelerating factor (DAF), CD59, complement receptor type 1 (CR1), factor I (FI), Vn and Cn) are included in the model, as well as the only known positive regulator, properdin (P), which can stabilize the C3 and C5 convertase.

We aimed at building a single model of the AP closely representing the biochemical pathway capable of reproducing in vitro and in vivo experimental observations. The structure of the proposed model is depicted in Fig 1. The model describes:

1. the known complement protein interactions in the fluid phase and their negative regulators (reaction 1–36 in S1 Appendix)
2. the association of complement proteins to erythrocytes, their interaction on the cell membranes together with their positive and negative regulators (reaction 37–111)
3. the hemolysis of erythrocytes as a function of AP activation (Eq 1)
4. the synthesis (reaction 114–129) and degradation (reaction 130–223, Eq 2) of complement proteins and their complexes in the human body
5. the physiological turnover of human erythrocytes, i.e. their natural production and elimination (reaction 112 and 113, Eq 3)
6. the kinetics of the hematological biomarkers, hematocrit (Eq 4), hemoglobin (Eq 5) and lactate dehydrogenase (LDH, Eq 6).

These individual modules are described in more detail below.

All simulations presented in this work are based on the same model. For prediction of experimental outcomes, the model parameters related to the experimental conditions were adjusted accordingly, while all the parameters intrinsic to the AP, such as the reactions rates, were kept constant. To simulate the in vitro experiments involving fluid phase proteins only, only module 1) was used. To simulate in vitro experiments involving rabbit or human erythrocytes, modules 1) to 3) were used. Details on each of the experimental setups and the respective implementation are provided below. All model equations and parameters are summarized in S1 and S2 Appendix, respectively. An overview of the literature sources used for model development and verification is provided in S1 Table.

#### Module 1 and 2 –Modelling of complement protein interactions

Complement protein interactions were modelled with a set of ODEs according to the law of mass action in case of association or dissociation of proteins, else as Michaelis-Menten kinetics for enzymatic reactions. Michaelis-Menten enzymatic reactions were parameterized with a catalytic rate constant k_cat_ and a Michaelis-Menten constant K_m_ (S1 Appendix and S2 Appendix) according to $\frac{d[P]}{dt}=k_{cat}[E]\frac{\left[S\right]}{K_{M}+[S]}$, where *[P]*, *[E]* and *[S]* are the concentration of the product, enzyme and substrate, respectively. It was assumed that the fluid phase is a well-mixed compartment without spatial concentration gradients.

The model by Zewde and coworkers [39] was used as a foundation for this work to investigate complement activation as measured in vitro in laboratory assays and in vivo via clinical markers of disease. To this end, the model was modified in several ways. The following reactions, assumed to occur physiologically [40], were included: dissociation of FH from C3bBbH_fluid_, C3bBbH_host_ and C3bBbC3bH_host_ (where “fluid” and “host” indicate fluid-phase and surface-bound complexes, respectively; reactions 35 and 104 in S1 Appendix); dissociation of CR1 from C3bBbCR1_fluid,_ C3bBbCR1_host_ and C3bBbC3bCR1_host_ (reactions 36, 106 and 107), dissociation of DAF from C3bBbDAF_host_ and C3bBbC3bDAF_host_ (reactions 108 and 109), and dissociation of properdin from C3bBbP_host_ and C3bBbC3bP_host_ (reactions 110 and 111). Additionally, conversion of C5 by the convertase was modelled as an enzymatic reaction (reactions 55 and 82), with the associated kinetic rate constants (*k*_*C*5,*cat*,*C*3*bBbC*3*b*(*P*)_ and *K*_*C*5,*m*,*C*3*bBbC*3*b*(*P*)_) obtained from the literature [43–45]. Binding kinetic parameters of FH (*k*_*p*,*C*3*bH*_ and *k*_*m*,*C*3*bH*_) and CR1 (*k*_*p*,*C*3*bCR*1_ and *k*_*m*,*C*3*bCR*1_) were obtained from [40,46]. Finally, the binding of nascent C3b with its reactive thioester to water forming fluid phase C3b was modelled as a first-order reaction with a half-life of 60 μs [47] (reactions 9 and 10 in S1 Appendix) instead of a second order reaction of binding of H_2_0 and nascent C3b. Further steps of model development are described in the following sections.

#### Module 3 –MAC-mediated lysis

**Fig 2:**
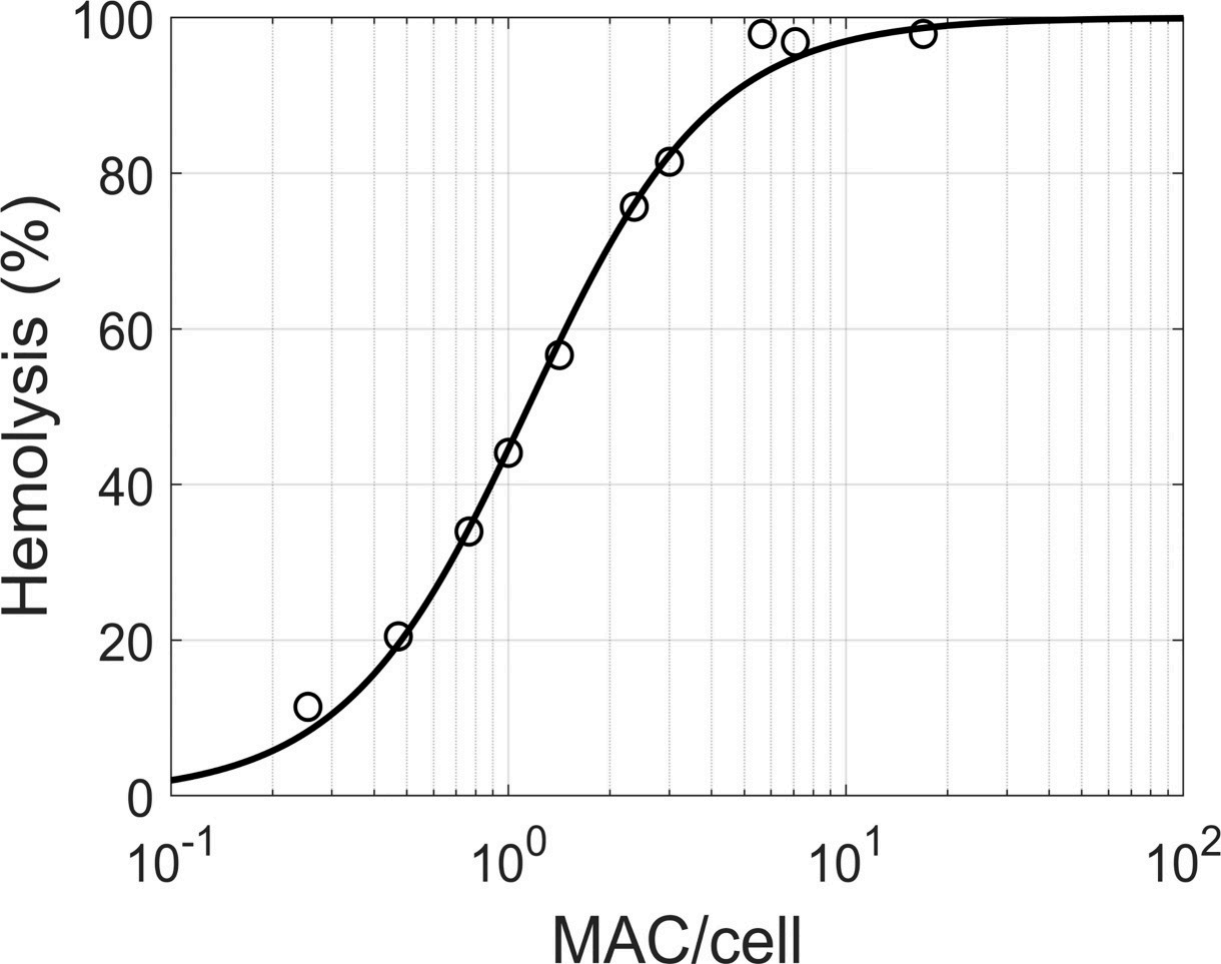
MAC-mediated hemolysis. Percent hemolysis displayed against the number of MACs per cell (module 3). Data (circles) of human complement-mediated lysis experiments on sheep erythrocytes were obtained from [48] (S3 Appendix). The data was fitted with the sigmoid model of Eq 1 (line), with *γ* = 1.60 and *MAC*50 = 1.15 MAC/cell.

The cytolytic effects of the MAC were included by modelling the relationship between MAC density and cell lysis (Eq 1). Takeda and colleagues [48] provided data on hemolysis of sheep erythrocytes carrying human C5b-7 complexes that were treated with fixed amounts of C8 and excess of C9. Based on this data, a dose-response curve for MAC-mediated lysis was obtained (Fig 2, S3 Appendix). We found that the percentage of cells lysed can be described by a sigmoid function of the number of MAC pores per cell, according to Eq 1 (Hill equation):

$$H=\frac{100}{1+(\frac{MAC50}{MAC}{)}^{\gamma }}$$ (1)

where *H* is the percent hemolysis, *MAC* the number of MACs per cell, *MAC*50 the number resulting in 50% hemolysis, and *γ* the Hill coefficient determining the steepness of the response. While there is experimental evidence that MAC complexes with less than 18 C9 molecules can lead to cell lysis [49], smaller MAC pores are likely less lytic than bigger MAC pores. Since there was insufficient experimental data to parameterize this relationship, it was assumed that only MACs complete with 18 C9 molecules [50] are functional structures capable of lysis.

#### Module 4 and 5 –Complement protein and erythrocyte turnover

In order to reproduce pathway homeostasis in the in vivo setting, the non-AP specific production and elimination of complement proteins was incorporated in the model such that the reported in vivo steady state concentrations of all AP molecules would be obtained at homeostasis. This included the synthesis of all components that are not a result of the AP reactions such as C3, FH, FB, FD, as well as the elimination through processes such as renal filtration, receptor mediated uptake or metabolism that are not part of the AP. These processes were not mechanistically modelled, instead production rates and first order elimination rates were used. The elimination rate constants of C3a, C5a, and C3dg were obtained from literature reports (S4 Table). Due to the limited availability of data, the clearance of the other complement proteins was estimated based on their molecular weight, assuming a sigmoidal relationship between the log-transformed hydrodynamic radius and the elimination rate constant (S4 Appendix). To constrain the number of independent parameters, fluid-phase C3 and C3b- and C5b-complexes were assumed to disappear at the same rate as C3 and C5, respectively. Considering that mature erythrocytes do not perform endocytosis [51], the elimination of surface-bound proteins and complexes was instead assumed to occur at the same rate as the cell itself, having a half-life in circulation of 60 days.

Rates of protein synthesis were estimated under the assumption that the AP of healthy individuals produces negligible complement-mediated hemolysis. The rate constants were calculated in order to match protein steady state levels (S2 Table) and elimination half-lives (S4 Table). The parameters controlling C3(H_2_O) hydrolysis ($k_{p,C3,H_{2}O}$) and the association of C3b to the surface (*k*_*p*,*C*3*b*,*surface*_) were decreased 10^2^- and 10^5^-fold relative to the in vitro parameterization, respectively, to compensate the increased pathway reactivity in vitro [52] and attain minimal terminal pathway activity in the absence of a trigger.

The elimination rate constant of cells and surface proteins, *k*_*el*,*S*_, was calculated according to Eq 2:

$$k_{el,S}=k_{s}+k_{H}$$ (2)

Here *k*_*s*_ describes the physiological turnover of erythrocytes and *k*_*H*_ the degradation due to hemolysis, as per Eq 3:

$$k_{H}=\frac{-ln(1-\frac{H}{100})}{\tau }$$ (3)

*H* being the percent hemolysis (Eq 1) and *τ* a scaling coefficient, which was estimated by fitting the hemoglobin levels measured in PNH type 3 patients as described below.

#### Module 6 –Hematological biomarkers

Several hematological markers are used clinically in the diagnosis and management of PNH disease. Hematocrit, hemoglobin and LDH levels provide relevant information for the monitoring of complement-mediated hemolysis and response to treatment. These markers were therefore included as part of the AP model. Hematocrit (*Hct*, %) was calculated according to Eq 4:

$$Hct=C_{e}*V_{e}*100\%$$ (4)

where *C*_*e*_ is the concentration of erythrocytes (5*10^6^ cells μL^-1^) and *V*_*e*_ the mean corpuscular volume (90 fL). Hemoglobin levels (*C*_*h*_, g dL^-1^) were calculated using Eq 5:

$$C_{h}=\frac{C_{e}*N_{h,e}*MW_{h}}{N_{A}}$$ (5)

where *N*_*A*_ the Avogadro constant, *N*_*h*,*e*_ the number of hemoglobin molecules per erythrocyte (assumed as 270*10^6^ cell^-1^ [53]) and *MW*_*h*_ their molecular weight (64.5 kDa [54]). Finally, LDH levels (*C*_*LDH*_, U L^-1^) were assumed to be a function of hemoglobin concentration according to Eq 6:

$$C_{LDH}=\frac{{LDH}_{max}}{1+exp(C_{h}-H_{LDH50})}+LDH_{0}$$ (6)

where *LDH*_*max*_, *H*_*LDH*50_, and *LDH*_0_ are parameters fitted to experimental data in PNH patients. While Eq 6 neglects the delay of LDH removal from circulation following cell lysis, it was found to describe the observations reasonably well (S1 Fig).

### Parameter estimation

**Fig 3:**
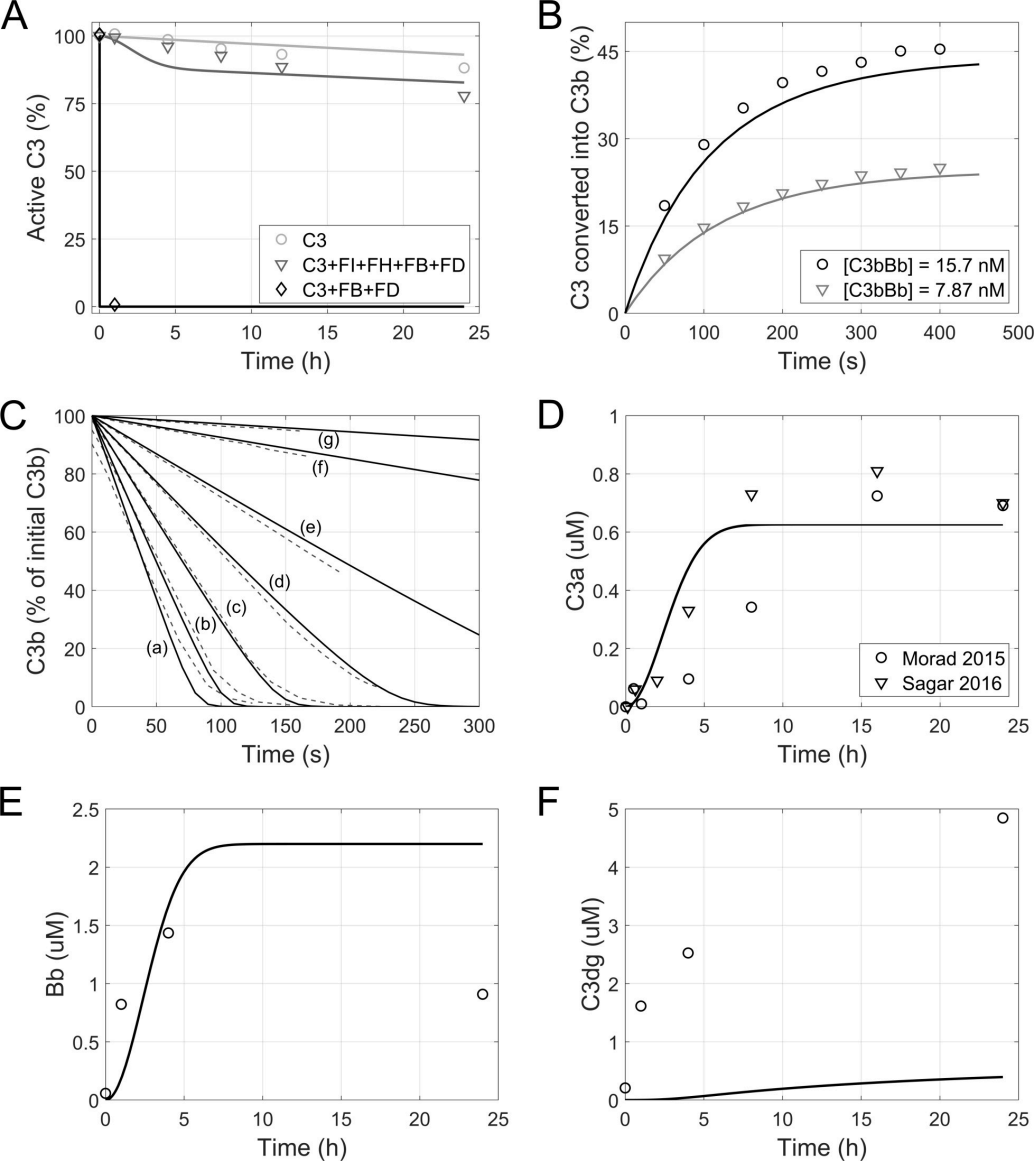
Fluid-phase alternative pathway activation. Observed (symbols) and simulated (lines, module 1) complement factor levels in experiments of fluid-phase AP activation in in vitro samples. (A) Inactivation of C3 in absence or presence of FB, FD, FH, and FI. (B) Formation of C3b by preassembled C3 convertases. (C) Conversion of C3b to iC3b as a function of FH concentration (790 nM (a), 393 nM (b), 197 nM (c), 98.5 nM (d), 49.3 nM (e), 12.4 nM (f), 4.50 nM (g). Dashed lines: experimental data, solid lines: model simulation). (D-F) Formation of activation markers C3a (D), Bb (E), and C3dg (F) due to spontaneous activation of AP in human serum samples. Data was obtained from [10] (A), [62] (B), [61] (C), [38,64] (D), and [63] (E,F). C3a levels from [38,64] were baseline corrected. Bb and C3dg levels from [63] were converted to molar concentrations for comparison to model results, assuming a molecular weight of 63 kDa and 38 kDa, respectively.

**Fig 4:**
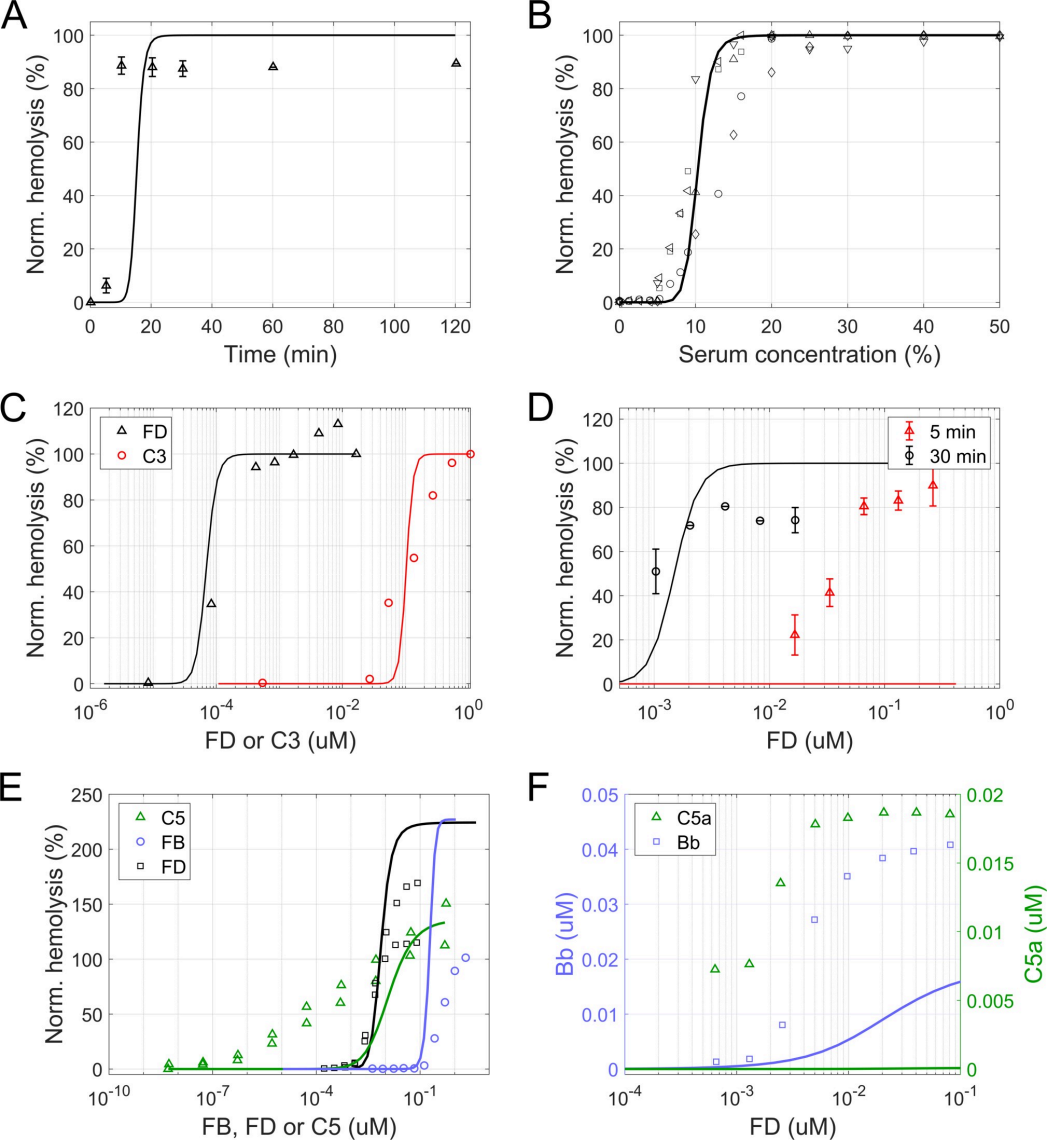
In vitro hemolytic experiments with rabbit erythrocytes. Experimental data (symbols) and model simulations (lines module 1–3) in hemolytic assays with rabbit erythrocytes. (A) Time course of hemolysis at 400 ng/mL FD. (B-E) Dose-response curves for hemolysis of rabbit erythrocytes. (B,C) Hemolysis at different serum dilutions (B) or when using a mix of normal human serum and FD- (black) or C3-depleted (red) serum (C). (D) Hemolysis as a function of FD serum concentration at 5 (red) and 30 minutes (black). (E) Hemolysis at different concentrations of C5 (green), FB (blue) and FD (black). For titration of C5 and FD, data from two individual experiments are shown. (F) Dose-response curve for activation markers Bb and C5a as a function of FD concentration. Hemolysis was normalized to lysis in water (A-D) or to the activity of NHS assayed in parallel at the same dilution (E). The experimental data was obtained from [56] (A,C,D), [55] (B), and [60] (E,F).

**Fig 5:**
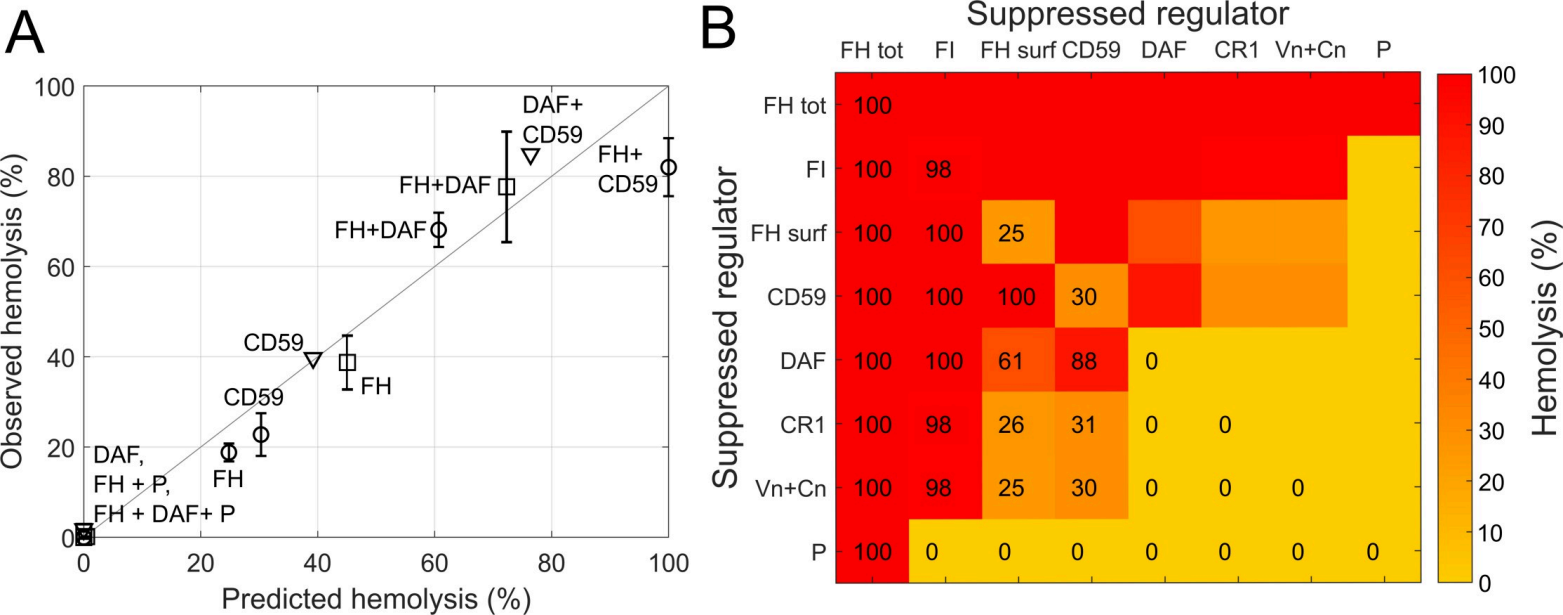
Hemolysis of human erythrocytes under partially disabled surface regulation. (A) Observed versus predicted (module 1–3) hemolysis of human erythrocytes. Observations are from [59] (triangles), [57] (circles) and [58] (squares). Inhibited regulators are indicated. Suppression of FH regulation was assumed to be restricted to the surface. (B) Model predicted (module 1–3) hemolysis for pairwise suppressed regulators. Suppressed regulators are indicated on the axes (FH surf: abolished FH surface regulation, FH tot: complete absence of FH regulation). Predicted hemolysis is shown as heat map and with percent values.

Data from fluid phase AP activation experiments (Fig 3) and rabbit (Fig 4) and human hemolytic assays (Fig 5) were split into an estimation and validation set. Model parameters were fitted by minimizing the sum of squared residuals (*SSR*) between simulated and experimental results of rabbit and human hemolytic assays [55–59] (Fig 4A–4D and Fig 5A). While data from the fluid phase AP activation experiments (Fig 3) and observations from Thanassi et al. [60] (Fig 4E and 4F) were used as an independent data set for model verification. A detailed list of the experiments and their use in the parameter estimation is given in S1 Table. For each experiment *j*, the *SSR* was calculated according to Eq 7:

$$SSR_{j}(x)=\frac{\sum_{i=1}^{n}(y_{i}-m_{i}(x){)}^{2}}{n}$$ (7)

where *y*_*i*_ is the *i*^th^ observation, *m*_*i*_(*x*) the model-predicted value with parameter value *x*, and *n* the number of data points. Normalization by the number of observations achieved equal weighing among studies. The objective function was defined as the sum of *SSR* for the five data sets:

$$SSR_{Sum}(x)=\sum_{j\epsilon S}SSR_{j}(x),$$ (8)

for *S* = {[55–59]}

*SSR*_*Sum*_(*x*) was minimized with respect to *x* using the *fminbnd* function in MATLAB R2018b, with options *TolX* and *TolFun* set to 0.01 and 0.5, respectively. Due to computational complexity, the parameters were optimized with an iterative approach. At each iteration, all parameters were individually fitted in a space ±7 orders of magnitude around the initial value; the parameter producing the lowest objective function value was updated in the model. The procedure was repeated until improvement in the objective function was below 1%. The choice of this approach is discussed below.

### Simulations

#### Fluid-phase alternative pathway activation

A fundamental assay of complement research involves the study of AP activation in the fluid phase by the spontaneous hydrolysis of C3. Various experimental setups have been described in the literature [10,38,61–64]. For simulation of these experiments, all surface-related species and reactions were excluded from the model (reaction 37–111 in S1 Appendix). For those experiments involving a subset of (recombinant) complement proteins, the initial concentration of fluid-phase species was set to the assay conditions reported in the respective publication. For simulations of AP activation in whole human serum samples, the initial conditions presented in S2 Table were used.

#### Rabbit erythrocyte lysis

Rabbit erythrocytes lack surface regulators of the human AP and rapidly undergo lysis when exposed to normal human serum. They are therefore often employed to perform in vitro studies of pathway activation against foreign cells. For instance, Pangburn and colleagues [55] investigated the lysis of rabbit erythrocytes at different serum dilutions. Wu et al. [56] and Thanassi et al. [60] reported dose-response curves of hemolysis for different levels of complement proteins C3, C5, FB, and FD. In order to achieve an accurate description of AP activity in vitro, we performed model simulations that reproduced these hemolytic assays in silico. In the simulations, rabbit erythrocytes were assumed to lack regulation by CR1, DAF, CD59, and FH [57,65–67]. The concentration of CR1, DAF, and CD59 were therefore set to 0, as well as the association rate constant for the binding of FH to surface-bound proteins (*k*_*p*,*C*3*bH*,*surf*_). Initial cell concentration, serum dilution and readout time were set to match the reported experimental conditions (S3 Table). Serum dilutions were modelled with equivalent reductions of the initial concentrations in 100% serum (S2 Table).

#### Human erythrocyte lysis

Human erythrocytes are protected against autologous complement-mediated lysis, nevertheless they may become susceptible if regulation is deficient, as is the case in PNH disease. To study the contribution of complement regulators, we collected literature data of hemolysis experiments performed with inhibitors [57–59] which we replicated in silico. In those studies, CR1, DAF, and CD59 were inhibited with antibodies. FH was blocked by FH19-20, a recombinant protein that inhibits FH cell-surface regulatory functions while preserving fluid-phase control of complement [57]. Due to limited information on the dose-response of inhibition, complete blockage was assumed in the simulations and the results were compared to the observations at the highest inhibitor concentration. The initial cell concentration and serum dilution used in the simulations matched the experimental conditions (S3 Table). The readout time was set to 30 minutes, a typical duration used in hemolytic experiments to allow sufficient pathway activation.

#### Alternative pathway in PNH

PNH is a disease resulting from impaired cell surface complement regulation. Deficiency of the regulatory proteins DAF and CD59 on erythrocyte membranes accounts for the intravascular hemolysis that is the clinical hallmark of the disease [15–17]. In order to study the behavior of the pathway in PNH patients, surface dysregulation was implemented in a model of disease. CD59 and DAF were assumed to be entirely absent from erythrocyte membranes in PNH type 3 patients. For type 2 patients, a 10-fold reduction in their synthesis rate was assumed, mimicking the subtotal deficiency (~10% of normal expression) that was determined in flow cytometric analyses [68]. The PNH clone size, namely the fraction of affected erythrocytes, was assumed to be 100%.

#### Eculizumab target inhibition and pharmacokinetics

To simulate the effects of eculizumab on hemolysis, a description of target engagement was included in the model (reaction 224 in S1 Appendix). The binding of eculizumab to its target C5 was described according to the law of mass action as:

$$C_{E}+C_{C5}⇋C_{E}C_{C5}$$ (9)

where *C*_*E*_, *C*_*C*5_, and *C*_*E*_*C*_*C*5_ are the eculizumab, C5, and drug-target complex concentration, respectively. The off-rate was calculated from the reported K_D_ value of 120 pM [69] and an assumed on-rate of 20 nM^-1^ day^-1^ based on the on-rates previously determined for 1500 antibodies [70,71]. Drug-bound C5 was assumed to be completely inhibited and functionally inactive.

In simulations of the inhibition of in vitro hemolytic activity, the initial cell concentration in the incubation, serum dilution, assay readout time, and eculizumab concentration were set to match the reported experimental conditions (S3 Table). Although PNH phenotype is known to differ among patients [68,72], type 2 erythrocytes were assumed due to the lack of donor information.

For the simulation of eculizumab treatment in PNH patients, the systemic pharmacokinetics were described with a one-compartmental model, assuming linear clearance (reaction 225 and 226 in S1 Appendix), an elimination half-life of 14.3 days, a molecular weight of 148 kDa, and a volume of distribution of 6.5 L [73]. Eculizumab dosing was recapitulated from [74] with eculizumab administered once weekly as an intravenous infusion of 600 mg for the first 4 weeks and 900 mg once every 2 weeks from week 5. Pre-treatment hemoglobin levels of the clinical study population [74] were matched adjusting the CD59 and DAF synthesis rates. The impact of transfusions was neglected.

#### Software

Data from publications was digitized with the *DigitizeIt* software (I. Bormann, *DigitizeIt* version 2.3.3, 2016. Retrieved from http://www.*digitizeit*.de/). ODEs were implemented and simulated in the SimBiology toolbox of MATLAB software version R2018b (MATLAB R2018b, The MathWorks, Inc., Natick, Massachusetts, United States). Unless stated otherwise, parameters were fitted using the lsqcurvefit function with default options as implemented in MATLAB R2018b.

## Results

### Parameter estimation

The final model consisted of 226 reactions, of which 111 described the AP, 112 described complement protein turnover and 3 were related to eculizumab. In total there were 121 parameters of which 70 were kinetic rate constants directly related to complement protein interactions and enzymatic reactions within the AP, 39 to non-AP related synthesis and elimination reactions, and 2 to eculizumab binding. The remaining 10 parameters were used to parameterize e.g. hemolysis and the dynamics of the hematological biomarkers. A preliminary assessment of model performance with the parameterization by Zewde et al. [39] showed inadequate agreement with experimental hemolysis data. Parameter fitting of the kinetic rate constants was thus performed. An iterative parameter estimation approach was chosen as to obtain a parameterization of the model that describes the experimental data with as few changes to the parameter values derived from the literature (S2 appendix) as possible. All 70 kinetic rate constants were included in the iterative parameter estimation approach, out of which only a small set (8) of sensitive parameters was optimized (Table 1, S2 Fig). Three of the parameters changed during the estimation are directly related to CD59 and DAF, increasing their contribution to pathway regulation. The others influence the positive regulation of the pathway (association of properdin to C3b, attachment of nfC3b to erythrocyte surface and association of FB to C3b), its initial activation in the fluid phase (cleavage of C3 by initial convertase C3(H_2_O)Bb), and the terminal activity (association of C6 to complex C3bBbC3bC5b). All results presented below were generated with the final model parameterization. Two of the final estimates were greater than the diffusion limit of protein-protein association of 10^9^ M^-1^s^-1^. These two parameters were linked to negative regulators of the pathway (association of CD50 to C5b9 and association of DAF to C3 convertase) occurring on the surface of erythrocytes. These reactions likely involve co-localization or clustering of inhibitors to pre-formed complement complexes and occur on the 2D cell surface rather than in 3D space and are therefore not limited by the diffusion limit.

**Table 1:** Parameters optimized in the fitting procedure.

Biochemical reaction	Parameter	Unit	Initial value	Final estimate
Attachment of nfC3b to erythrocyte surface	*k*_*p*,*C*3*b*,*surface*_	M^-1^s^-1^	4.2*10^8^	2.16*10^9^
Association of CD59 to C5b9	*k*_*p*,*CD*59*C*5*b*9_	M^-1^s^-1^	1.0*10^6^	6.03*10^11^
Association of properdin to C3b	*k*_*p*,*C*3*bP*_	M^-1^s^-1^	3.0*10^6^	1.24*10^8^
Association of DAF to C3 convertase	*k*_*p*,*C*3*bBbDAF*_	M^-1^s^-1^	2.0*10^3^	2.53*10^10^
Cleavage of C3 by C3 convertase C3(H20)Bb	$K_{C3,m,C3(H_{2}O)Bb}$	M	5.9*10^−6^	4.19*10^−6^
Decay of C3 convertase by inhibitor DAF on host cell	*k*_*m*,*C*3*bBbDAF decay*_	s^-1^	7.7*10^−2^	2.28*10^−3^
Association of C6 to C3bBbC3bC5b	*k*_*p*,*C*3*bBbC*3*bC*5*bC*6_	s^-1^	6.0*10^4^	7.74*10^4^
Association of FB to C3b	*k*_*p*,*C*3*bB*_	M^-1^s^-1^	2.13*10^5^	2.23*10^5^

S2 Fig shows the result of a local sensitivity analysis that was conducted with respect to the *SSR*_*Sum*_ (Eq 8). At the optimum identified by the parameter estimation approach some of the optimized parameters were insensitive to changes of ±20% while they significantly contributed to the improve in the objective function during parameter estimation (S2 Fig). Other parameters, such as binding of FH to C3b on the other hand, were highly sensitive but were not changed during the parameter estimation approach. The reason for this might be that the initial guess of these parameter values was already close to optimal.

### In vitro alternative pathway activation and hemolytic activity

#### Fluid-phase alternative pathway activation

Upon simulation of various in vitro assays with purified complement proteins and whole serum samples, the model was found to be in good agreement with experimental finding. It reproduced the baseline inactivation of purified C3 and the changes determined by the addition of purified FB and FD, in the presence or absence of FH and FI (Fig 2A). The model accurately captured the conversion of C3 into C3b by pre-formed C3 convertase (Fig 2B) and the conversion of C3b to iC3b in the presence of different concentrations of FH (Fig 2C). The model also replicated the spontaneous AP activation in whole human serum samples, largely predicting the kinetics and steady state levels of C3a and Bb (Fig 2D and 2E). An underprediction was observed for the accumulation of C3dg (Fig 2F). It should be noted that C3dg formation was measured after serum activation with heat-aggregated immunoglobulin G, which also triggers the classical pathway [63]. Due to the unavailability of C3dg results specific to AP activation, this data set was nevertheless used for model validation.

#### Rabbit erythrocyte lysis

In simulations of the lysis of rabbit erythrocytes at different serum dilutions, the model was able to reproduce the rapid kinetics of hemolysis (Fig 4A) as well as the dependence on serum concentration (Fig 4B). It was also able to replicate the findings by Wu et al. [56] that rabbit erythrocyte lysis is induced by significantly lower concentrations of FD as compared to C3 (Fig 4C). When comparing the predicted dependency on FD at 5 and 30 minutes after the start of incubation, the model reproduced the sensitivity of the pathway at 30 minutes but did not predict any lysis at 5 minutes, contrary to the observations (Fig 4D). This showed that the model overpredicted the time to onset of hemolysis, as also seen in Fig 4A. S4 Fig shows a local sensitivity analysis of the pathway activation as measured by the formation of MAC complexes on rabbit erythrocytes in a standard rabbit erythrocyte hemolysis experiment to the kinetic rate constants and initial concentrations of AP proteins. The most sensitive parameters were parameters controlling the initial pathway activation such as association of FH or FB to C3b or the association of C3b to the erythrocyte surface. Most parameters related to the terminal pathway on the other hand were relatively insensitive within the tested range of ±20%. Similarly, the most sensitive initial concentrations were found to be C3, FB, FD and FH indicating that in the simulated scenario pathway activation is not limited by the terminal pathway such as e.g. availability of C9.

To verify the predictivity of the model with data not included in the fitting procedure, we simulated the hemolytic experiments described by Thanassi and co-workers [60]. The model accurately predicted pathway activation in response to FD and, at higher concentrations, FB (Fig 4E). Interestingly, the concentration-response curve for FD was significantly steeper than that reported by Wu et al. [56] (Fig 4C) and a closer match to the model prediction. In the experimental setup, hemolysis was normalized to the activity of normal human serum assayed in parallel at the same dilution (8.3%). The model underpredicted lysis at this dilution, leading to an overprediction of the maximal normalized hemolysis. In terms of C5 and the activation markers Bb and C5a (Fig 4E and 4F), the model failed to reproduce the experimental observations. Here, it is noteworthy that at least determining C5a can be highly challenging due to C5a being generated during test tube handling [75,76]. As a result, the actual concentration of C5a might be lower than the measured value.

#### Human erythrocyte lysis

In experiments with human erythrocytes, it was shown that inhibition of CD59 or FH surface regulation results in significant lysis (up to 40% in 30 minutes), while inhibition of DAF does not produce any hemolysis [57,59]. The model adequately replicated these findings as well as the effects of combined inhibition of FH and DAF, FH and CD59, or CD59 and DAF (Fig 5A). The model also replicated the experimental observation that the positive regulation by properdin is necessary for hemolysis [58].

We simulated the pairwise inhibition of AP regulators following the experimental setup of Ferreira and co-workers [57] (Fig 5B). The model predicted FH and FI to be the most important regulators, resulting in 100% and 98% hemolysis if absent. Abolished CD59 and FH surface regulation was predicted to result in 30% and 25% hemolysis, respectively. If their absence was combined with DAF suppression, predicted hemolysis increased to 88 and 61%. CR1, Vn, and Cn suppression was predicted to have little impact if combined with inhibited FI or FH surface regulation. Finally, no hemolysis was predicted in the absence of properdin, except when combined with FH suppression.

#### Eculizumab effects on lysis of PNH erythrocytes

**Fig 6:**
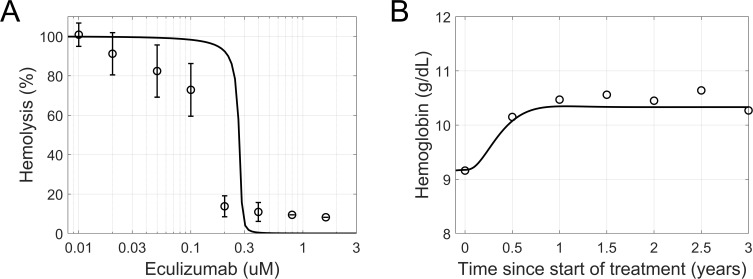
In vitro and in vivo effects of eculizumab in PNH disease. (A) Observed (symbols) and simulated (line, module 1–3) inhibition of PNH erythrocyte lysis in vitro as a function of eculizumab concentration. (B) Observed (symbols) and simulated (line, module 1–5) recovery of hemoglobin levels in PNH patients treated for 36 months with eculizumab. The experimental data was digitized from [77] (A) and [74] (B).

Replicating in vitro measurements of PNH erythrocyte lysis inhibition by eculizumab in acidified serum [77], the model captured the sensitive concentration range observed experimentally (Fig 6A). The simulations indicated a half maximal inhibitory concentration (IC50) of circa 0.3 μM, slightly overpredicting the experimental result (between 0.1 and 0.2 μM).

### In vivo alternative pathway homeostasis and pharmacology

#### Non-AP specific complement protein turnover

The synthesis rates of complement proteins, estimated assuming negligible activation in healthy individuals, and the corresponding fractional catabolic rates (FCR) are reported in S4 Table. The rates agreed reasonably well with previous estimates, for instance displaying a 2-fold deviation or less for FH, FD, C3, C5, and C9. FD exhibited the highest FCR among complement proteins, in agreement with previous reports. The largest discrepancies pertained to the synthesis rates of FB and properdin (6–10-fold higher than reported in the literature).

#### Hematological biomarkers

The hemoglobin levels in PNH type 3 patients were used to parameterize the rate of MAC-mediated hemolysis. The predicted LDH and hematocrit levels in PNH type 2 patients and healthy individuals matched very well the ranges reported in the literature (Table 2).

**Table 2:** Hematological biomarkers in healthy and PNH population.

Parameter	Unit	Status	Study	Source	Simulated^a^
Hemoglobin	g dL^-1^	Healthy	12–17		14
		PNH	6–10	[78]	4–11
LDH	U L^-1^	Healthy	100–190		300
		PNH	500–5000	[78,79]	380–1760
Hematocrit	%	Healthy	37–52		45
		PNH	20	[80]	13–34

^a^ Simulated ranges in PNH correspond to PNH type 2–3 (upper and lower boundary, respectively).

#### Eculizumab effects in PNH disease

We explored the in vivo effects of eculizumab on the hemoglobin levels in PNH patients (Fig 6B). The model predicted a slow and sustained recovery of hemoglobin, reaching a new steady-state level after about one year of treatment, as Choi and colleagues [74] observed in patients. The extent of recovery was less than the mean clinical response, however the variability of the observations is unavailable to help qualify this underprediction. Also, the model did not include the effect of transfusions.

## Discussion

Complement is becoming increasingly recognized as a major driver of several common and rare diseases such as PNH, aHUS and AMD [13] affecting millions worldwide. The large complexity of the biochemical reactions involved in complement homeostasis and activation makes it difficult to predict the behavior of the system in health and disease. Recognizing the need for an efficient tool to study AP biochemistry and pharmacology, we set out to construct a mathematical model of the pathway spanning from the tick-over mechanism of C3 hydrolysis to MAC formation (Fig 1). To this end, we took advantage of previous modelling efforts and experimental and clinical results of complement research available in the scientific literature to build and verify the model. Parameter estimation was performed by fitting model simulations to an ensemble of experimental observations, allowing us to match or approximate the results obtained by different groups under various conditions. The model was used to predict the in vitro and in vivo effects of a complement-targeting treatment, eculizumab, and the associated changes in functional and clinical markers of hemolysis. Thereby, we demonstrated how the model may be used to support complement research and drug development both in the nonclinical and clinical setting.

The model herein presented builds upon previous works offering a mechanistic description of the biochemical interactions involved in AP regulation and activation, in particular the work of Zewde et al. [39]. We extended previous approaches by including MAC-mediated cell lysis as a result of terminal pathway activation (Fig 2). This allowed us to replicate in model simulations the hemolytic experiments that are routinely used to study complement activation in vitro (Fig 4). Also, the in vivo turnover of complement proteins was incorporated in the model to mimic pathway homeostasis. PNH disease and the related changes in hemolytic markers were hence reproduced (Fig 6) by implementing the dysregulation of complement on erythrocyte membranes.

In our efforts to study the behavior of the pathway through in silico predictions, we first honed in on a well characterized step of the AP cascade: the spontaneous hydrolysis of the thioester group of the C3 protein. This mechanism represents the initial step in AP activation, followed by the formation of C3 proconvertase and convertase complexes and the concomitant cleavage of C3 into C3a and C3b. It has been described that pre-incubation of serum samples at 37°C causes a time-dependent decrease of hemolytic activity [10]. This effect is due to the spontaneous activity of the AP in the fluid phase, which leads to the consumption of the available C3 pool in the sample. In various settings involving the presence or absence of negative and positive regulators, the model correctly described AP activation in the fluid phase as reported by multiple sources [38,61,63,64] (Fig 2). It captured accurately the formation of key markers of pathway activation, C3a, Ba, and Bb, as well as the dynamics of C3 inactivation and the concurrent formation of C3b. This may prove useful for predicting the modulation of spontaneous AP activation upon targeting complement proteins, such as C3 (e.g. the drug candidate APL-2, Apellis Pharmaceuticals), FB (FB-LRx, Ionis Pharmaceuticals), or FD (ACH-0144471, Achillion Pharmaceuticals). Additionally, it may aid in the stratification of patients with differences in the plasma concentration of these complement proteins.

Next, we turned to investigate stimulated activation of the pathway. The AP hemolytic assay (AH50) is a frequently used screening test for complement abnormalities, and one of the standard assays to establish dose-response relationships in clinical trials. This method relies on the hemolysis of unprotected rabbit erythrocytes upon the addition of human serum. The model largely reproduced the rapid kinetics of hemolysis as well as the degree of lysis as a function of the concentration of serum and various complement factors (Fig 4A–4E). We found however an underprediction of the sensitivity to C5 (Fig 4E) and of C5a and Bb production at different FD concentrations (Fig 4F). It is noteworthy that C5a measurements are often assay-dependent [75,76]. Additionally, most of the functional complement assays use serum, because anticoagulants chelate divalent cations and the presence of magnesium ions is an absolute requirement for all three complement pathways. On the other hand, the interplay between proteases activated during coagulation/fibrinolysis and the complement system has long been observed and may explain the differences between predicted and measured values [81]. Another reason for the discrepancy could be the density-dependency of the enzymatic activity of the C5 convertase. Rawal and Pangburn [44] reported that the Michaelis constant *Km* decreased up to ~28-fold with an increase in density of C5 convertases on the cell surface, a mechanism not included in the present version of the model. While developing the model, we also found that the time of readout is a critical parameter in the model, as observed in the underprediction of the 5-minute lysis experiment (Fig 4D). Here it should be noted that due to the reactivity of the pathway, the time to lysis might be strongly impacted by the experimental conditions, which could not be fully controlled in the simulations (e.g. for the influence of sample handling or storage conditions). Finally, we considered a set of experimental studies [57–59] investigating the lysis of human erythrocytes with partially disabled surface regulation. The model adequately reproduced the hemolysis resulting from inhibition of surface regulators, individually and in combination (Fig 5). In sum, these results give confidence that the model can be used to predict the outcome of in vitro assays of complement activation and hemolysis.

Even though in vitro experiments are helpful to characterize drug candidates, the success of a treatment ultimately depends on its efficacy in vivo. We therefore adapted the model to reproduce the in vivo setting by incorporating the turnover (synthesis and elimination) of complement proteins so as to mimic AP homeostasis. Background activity is known to differ, the pathway being more reactive or partly activated in vitro. In fact, sample processing and assay conditions have been shown to influence the level of complement proteins significantly. For instance, it has been reported that thawing blood samples for one hour at room temperature versus at 37°C can lead to an over 200-fold increase [52]. Also, the use of MgEGTA or acidified serum in the assays results in AP activation [59,77,82,83]. Therefore, in order for the model to match the activity of the pathway in healthy individuals (Table 2), the value of the parameters regulating C3 tick-over ($k_{p,C3,H_{2}O}$) and C3b surface attachment (*k*_*p*,*C*3*b*,*surface*_) were reduced. The FB synthesis rate remained nonetheless 7-fold faster than reported in literature (S4 Table), suggesting that the model over-predicts the background activity in healthy individuals.

To establish the clinical applicability of the AP model, we examined PNH disease as a case study. In PNH, erythrocytes lack plasma membrane proteins imparting protection against complement attack, a defect that leads to chronic hemolysis. Hematological laboratory values such as hemoglobin, hematocrit, and LDH levels are therefore used in the clinical management of the disease. The development of eculizumab, a monoclonal antibody directed against C5, has resulted in dramatic improvements of survival and reduction in complications. The model was shown to predict the baseline levels of common hematological markers (hemoglobin, LDH, and hematocrit) in healthy subjects and PNH patients (Table 2), the effects of eculizumab in hemolytic assays with PNH erythrocytes (Fig 6A) and to match the recovery of hemoglobin observed in PNH patients under treatment (Fig 6B). We envision that in the future, the model could be parameterized so as to match the AP activity as well as PNH type and clone size of individual patients. This would represent a useful tool for promoting personalized healthcare in PNH, where dosing regimens could be individualized based on a patient’s inherited and acquired genetic makeup. On the other hand, a potential limitation of the present study is the neglect of extravascular hemolysis. Eculizumab has no effect on complement activity upstream of C5, such as the deposition of C3b on the erythrocyte membrane. Recognition of this signal by macrophages results in hemolysis that may reduce the clinical benefits. This limitation should be addressed in a future development of the model.

In conclusion, the model herein presented can reproduce in vitro and in vivo AP activation and AP-mediated hemolysis. It provides a mechanistic and quantitative framework to guide the design of studies and integrate experimental and clinical data. It may prove useful for testing novel therapeutic hypotheses, namely selecting targets for pharmacological intervention and predicting the efficacy of drug candidates. While we believe the model is a state-of-the-art representation of AP biochemistry, future studies might indicate that the model assumptions and parameterization should be revisited. The model could also be extended to other cell types, such as bacteria, allowing the use of a wider set of experimental information. Learn-and-confirm cycles between simulation and experimentation can help identify gaps in our understanding of the pathway and lead to future improvements in the model.

## Supporting information

S1 Fig LDH levels in PNH patients treated with eculizumab. Reported measurements of blood LDH and hemoglobin levels in 30 PNH patients treated with eculizumab (symbols) [79]. LDH levels were assumed to be a function of hemoglobin concentration according to Eq 6 (line). The parameters were fitted to the experimental observations prior to and during, after a minimum of 6 months from initiation of treatment, pharmacological treatment: *LDH*_*max*_ = 1495 U L^-1^, *H*_*LDH*50_ = 7.94 g dL^-1^, and *LDH*_0_ = 296 U L^-1^.
(TIF)

S2 Fig Convergence of model fit to experimental hemolysis data. The iterative optimization of parameters led to successive improvements in the objective function and an increasing convergence between model and data. The figure shows the fitted parameter at each iteration together with the associated change in sum of squared residuals (*SSR*_*Sum*_, Eq 8). The estimation procedure reached a stable objective function value (<1% change) after 10 iterations.
(TIF)

S3 Fig Local parameter sensitivity analysis with respect to *SSR*_*Sum*_. Kinetic rate constants were decreased (blue) or increased (red) by 1, 5, 10 and 20% (light to dark) around their final estimate and the change in *SSR*_*Sum*_ relative to the one obtained with the final parameterization was calculated. Boxes indicate parameters that were optimized during the iterative parameter optimization (Table 1, S2 Fig).
(TIF)

S4 Fig Local parameter sensitivity analysis with respect to terminal pathway activation. Kinetic rate constants and initial concentrations were decreased (blue) or increased (red) by 1, 5, 10 and 20% (light to dark), and the change of terminal pathway activation as quantified by MAC formation in a standard rabbit erythrocyte hemolysis assay was calculated. Experimental parameters used were 30 min readout time, 20% serum and 1*10^11^ cells/L which corresponds to a commonly used experimental setup (S3 Table).
(TIF)

S1 Table Literature sources used to inform model development. Description of literature sources and their use in model development.
(PDF)

S2 Table Initial concentration of model species used in model simulations. Initial conditions of the differential equations describing the change in protein concentrations over time. For in vivo simulations, these initial values coincide with the steady state levels. For unlisted proteins and complexes, the initial concentration was assumed equal to 0 or to their steady state level when simulating the in vitro or in vivo setting, respectively.
(PDF)

S3 Table Experimental conditions used for simulation of hemolytic experiments with rabbit and human erythrocytes. Cell species, publication, initial cell concentration, readout time and serum concentration for each of the simulated hemolytic experiments.
(PDF)

S4 Table Synthesis rate and elimination half-life of complement proteins used for in vivo simulations. Estimation of the systemic half-life of complement proteins based on their molecular weight as described in S4 Appendix.
(PDF)

S1 Appendix Alternative pathway model reactions. List of biochemical reactions of the AP, complement protein turnover reactions and reactions involving eculizumab.
(PDF)

S2 Appendix Alternative pathway model parameters. List of model parameters.
(PDF)

S3 Appendix MAC-mediated hemolysis. Derivation of quantitative relationship between MAC formation and hemolysis.
(PDF)

S4 Appendix Plasma elimination half-life of complement proteins. Quantitative relationship between molecular size and half-life.
(PDF)
