# Supplementary figures and images for: Modeling the activation of the alternative complement pathway and its effects on hemolysis in health and disease
Source: PLoS Comput Biol. 2020 Oct 2;16(10):e1008139. doi: 10.1371/journal.pcbi.1008139 (PMC7531836; doi:10.1371/journal.pcbi.1008139)

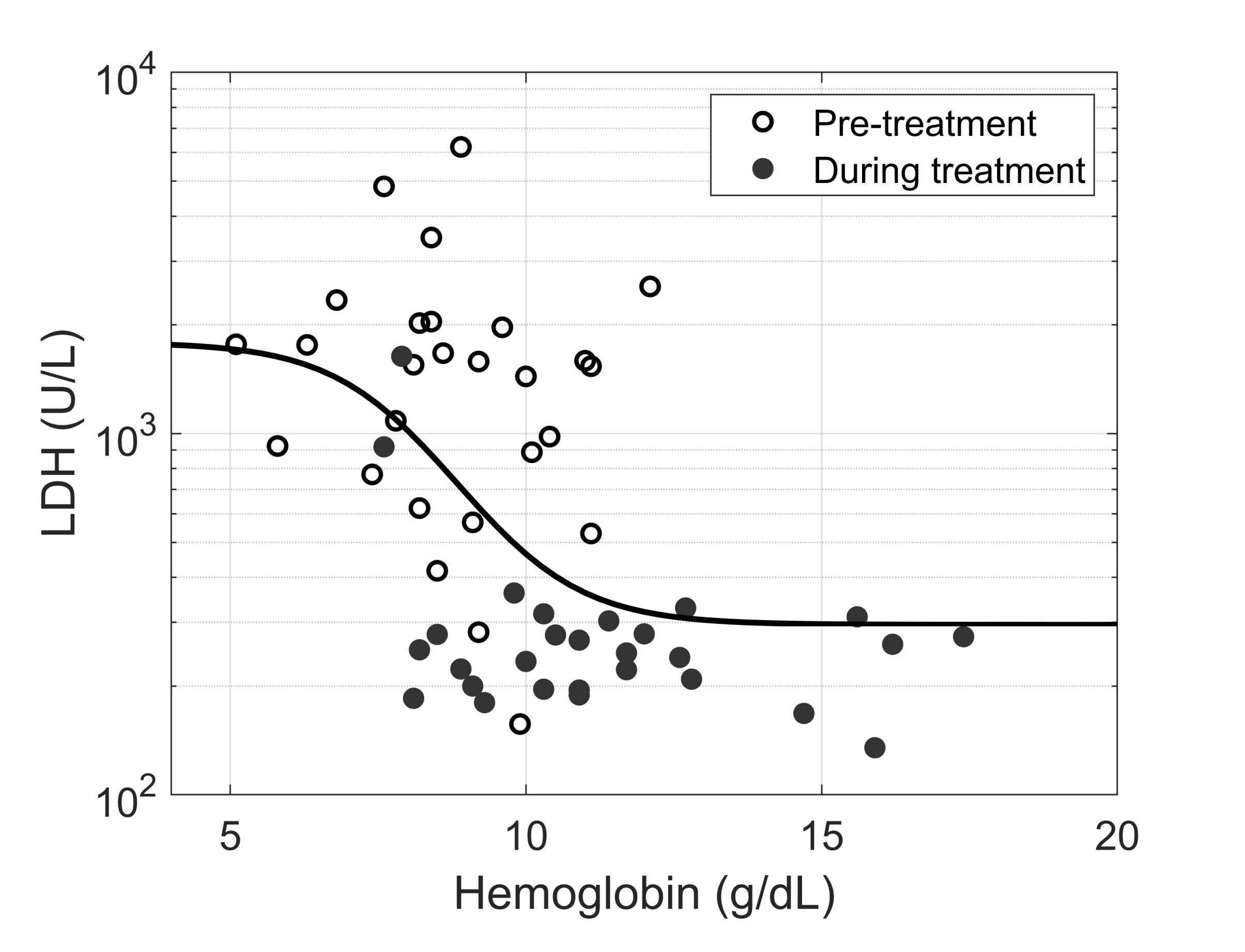

Supplement: S1 Fig — Reported measurements of blood LDH and hemoglobin levels in 30 PNH patients treated with eculizumab (symbols) [79]. LDH levels were assumed to be a function of hemoglobin concentration according to Eq 6 (line). The parameters were fitted to the experimental observations prior to and during, after a minimum of 6 months from initiation of treatment, pharmacological treatment: LDHmax = 1495 U L-1, HLDH50 = 7.94 g dL-1, and LDH0 = 296 U L-1. (TIF) [file pcbi.1008139.s001.tif]

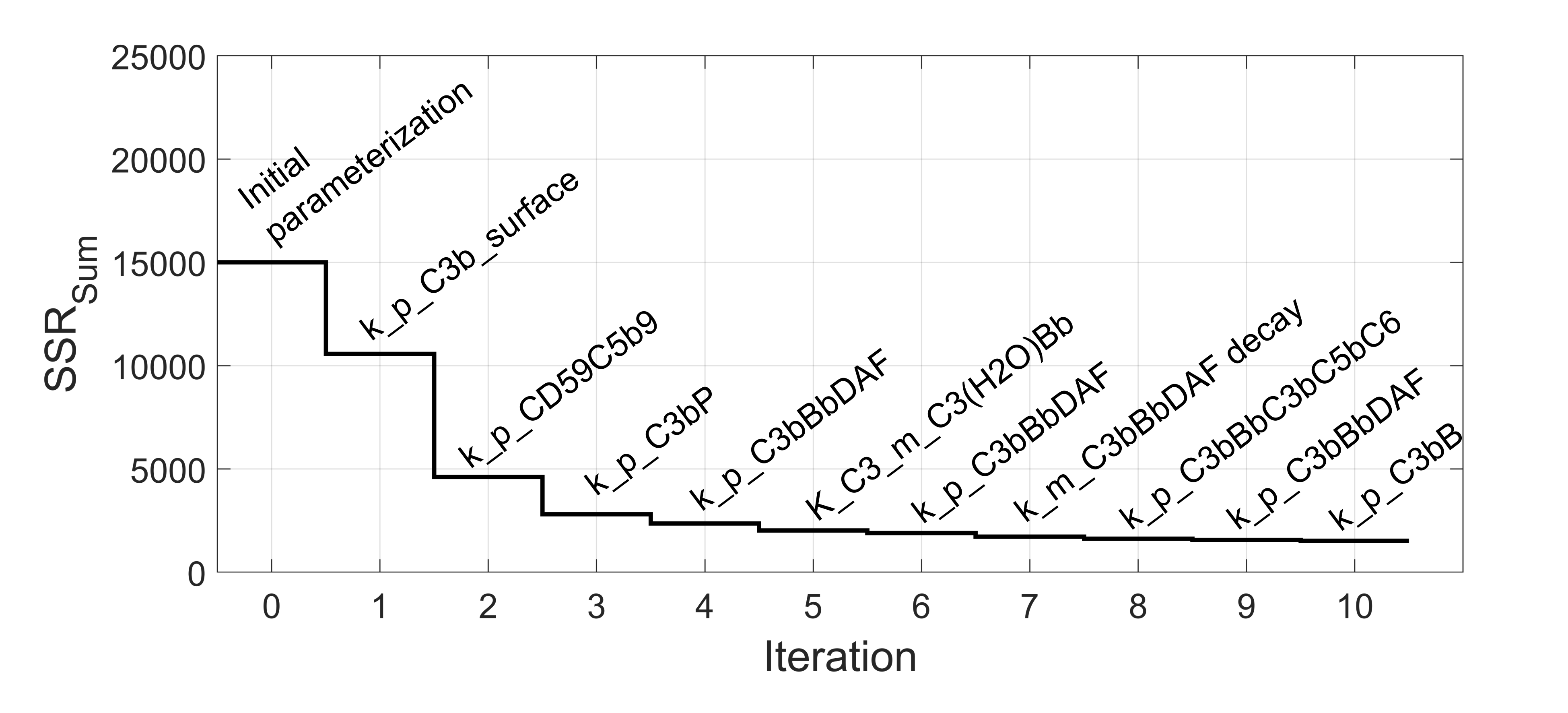

Supplement: S2 Fig — The iterative optimization of parameters led to successive improvements in the objective function and an increasing convergence between model and data. The figure shows the fitted parameter at each iteration together with the associated change in sum of squared residuals (SSRSum, Eq 8). The estimation procedure reached a stable objective function value (<1% change) after 10 iterations. (TIF) [file pcbi.1008139.s002.tif]

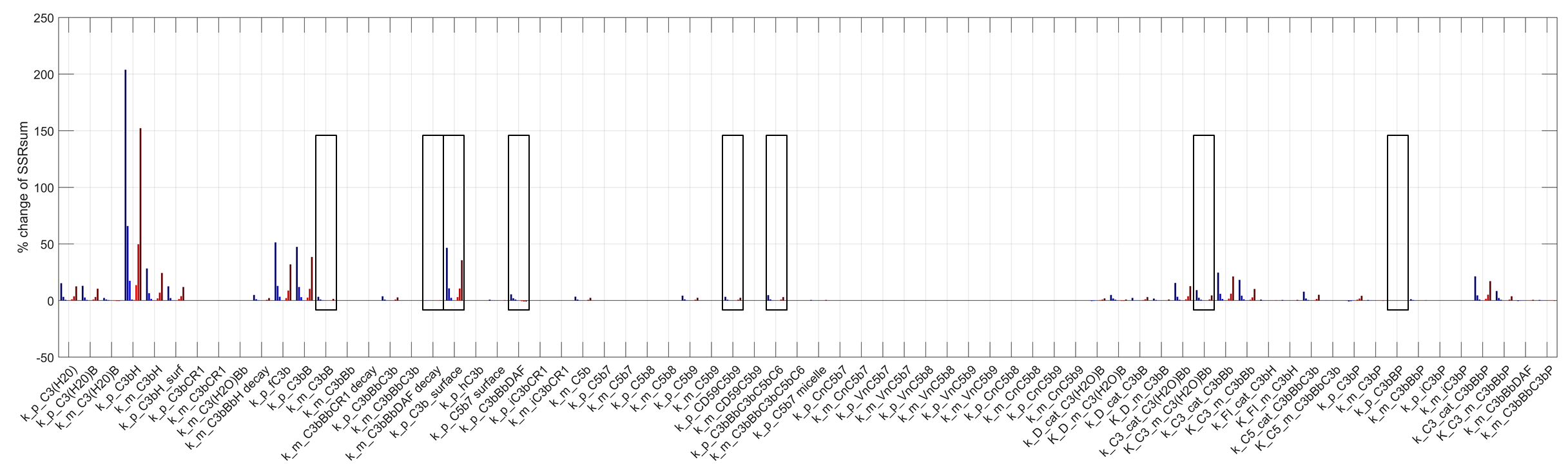

Supplement: S3 Fig — Kinetic rate constants were decreased (blue) or increased (red) by 1, 5, 10 and 20% (light to dark) around their final estimate and the change in SSRSum relative to the one obtained with the final parameterization was calculated. Boxes indicate parameters that were optimized during the iterative parameter optimization (Table 1, S2 Fig). (TIF) [file pcbi.1008139.s003.tif]

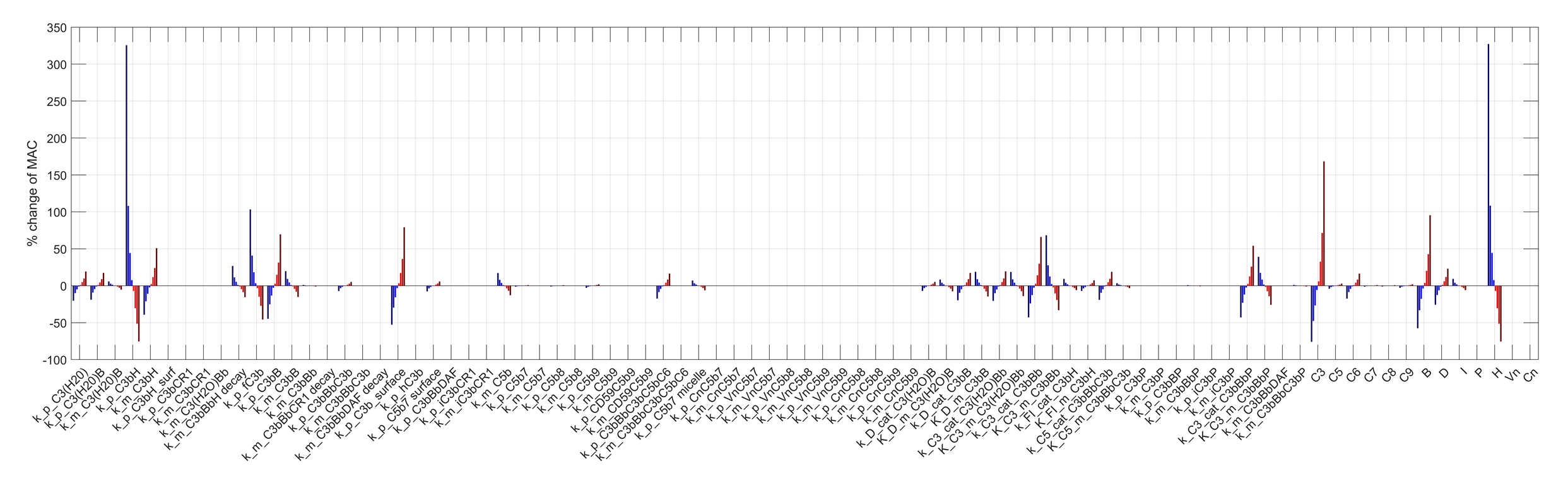

Supplement: S4 Fig — Kinetic rate constants and initial concentrations were decreased (blue) or increased (red) by 1, 5, 10 and 20% (light to dark), and the change of terminal pathway activation as quantified by MAC formation in a standard rabbit erythrocyte hemolysis assay was calculated. Experimental parameters used were 30 min readout time, 20% serum and 1*1011 cells/L which corresponds to a commonly used experimental setup (S3 Table). (TIF) [file pcbi.1008139.s004.tif]
