# Supplementary material for: Modeling the activation of the alternative complement pathway and its effects on hemolysis in health and disease
Source: PLoS Comput Biol. 2020 Oct 2;16(10):e1008139. doi: 10.1371/journal.pcbi.1008139 (PMC7531836; doi:10.1371/journal.pcbi.1008139)
Supplement: S1 Table — Description of literature sources and their use in model development. (PDF) [file pcbi.1008139.s005.pdf]

Description	Source	Experimental setting	Species	Used for parameter estimation	Figure
Fluid-phase inactivation of C3 in presence and absence of FI, FH, FB, FD	[1]	In vitro	Human proteins	No	2A
C3 conversion to C3b by C3bBb	[2]		Human proteins	No	2B
Cleavage of C3b by fixed concentrations of FI and FH	[3]		Human proteins	No	2C
Spontaneous C3a formation in serum samples	[4]		Human serum	No	2D
Spontaneous C3a formation in serum samples	[5]		Human serum	No	2D
Spontaneous Bb and C3dg formation in serum samples	[6]		Human serum	No	2E,F
Linking terminal pathway activity to hemolysis	[7]		Various	No	3
Time course of rabbit erythrocyte lysis	[8]		Rabbit cells, human serum	Yes	4A
Rabbit erythrocyte lysis as a function of FD and C3 titration and of FD titration at two different time points					4C,D
Rabbit erythrocyte lysis at different serum concentrations	[9]		Rabbit cells, human serum	Yes	4B
Rabbit erythrocyte lysis as a function of C5, FB, FD titration	[10]		Rabbit cells, human serum	No	4E
Bb and C5a production due to rabbit erythrocyte lysis					4F
Human erythrocyte lysis with inhibition of FH, DAF, CD59	[11]		Human	Yes	5A
Human erythrocyte lysis with inhibition of FH, DAF, properdin	[12]		Human	Yes	5A
Human erythrocyte lysis with inhibition of CD59, DAF	[13]		Human	Yes	5A
Human PNH erythrocyte lysis as a function of eculizumab titration	[14]		Human	No	6A
Recovery of hemoglobin in patients with PNH on eculizumab treatment	[15]	In vivo	Human	No	6B

References

1. Pangburn MK, Schreiber RD, Müller-Eberhard HJ. Formation of the initial C3 convertase of the alternative complement pathway. Acquisition of C3b-like activities by spontaneous hydrolysis of the putative thioester in native C3. J Exp Med. 1981;154: 856–67.
2. Pangburn MK, Müller-Eberhard HJ. The C3 convertase of the alternative pathway of human complement. Enzymic properties of the bimolecular proteinase. Biochem J. 1986;235: 723–30.
3. Pangburn MK, Müller-Eberhard HJ. Kinetic and thermodynamic analysis of the control of C3b by the complement regulatory proteins factors H and I. Biochemistry. 1983;22: 178–85.
4. Morad HOJ, Belete SC, Read T, Shaw AM. Time-course analysis of C3a and C5a quantifies the coupling between the upper and terminal Complement pathways in vitro. J Immunol Methods. 2015;427: 13–8.
5. Sagar A, Dai W, Minot M, LeCover R, Varner JD. Reduced order modeling and analysis of the human complement system. PLoS One. 2017;12: e0187373.
6. Bergseth G, Ludviksen JK, Kirschfink M, Giclas PC, Nilsson B, Mollnes TE. An international serum standard for application in assays to detect human complement activation products. Mol Immunol. 2013;56: 232–9.
7. Takeda J, Kozono H, Takata Y, Hong K, Kinoshita T, Sayama K, et al. Number of hits necessary for complement-mediated hemolysis. Microbiol Immunol. 1986;30: 461–8.
8. Wu X, Hutson I, Akk AM, Mascharak S, Pham CTN, Hourcade DE, et al. Contribution of Adipose-Derived Factor D/Adipsin to Complement Alternative Pathway Activation: Lessons from Lipodystrophy. J Immunol. 2018;200: 2786–2797.
9. Pangburn MK. Cutting edge: localization of the host recognition functions of complement factor H at the carboxyl-terminal: implications for hemolytic uremic syndrome. J Immunol. 2002;169: 4702–6.
10. Thanassi JA, Dharaben P, Yang G, Galvan M, Yhao Y, Fabrycki J, et al. Comparison of complement functional assays: Differential sensitivities of hemolysis and Wieslab assays to levels of complement proteins C5, factor B, and factor D. XXVI ICW Kanazawa. 2016. pp. 1035–1226.
11. Ferreira VP, Pangburn MK. Factor H mediated cell surface protection from complement is critical for the survival of PNH erythrocytes. Blood. 2007;110: 2190–2.
12. Lesher AM, Zhou L, Kimura Y, Sato S, Gullipalli D, Herbert AP, et al. Combination of factor H mutation and properdin deficiency causes severe C3 glomerulonephritis. J Am Soc Nephrol. 2013;24: 53–65.
13. Wilcox LA, Ezzell JL, Bernshaw NJ, Parker CJ. Molecular basis of the enhanced susceptibility of the erythrocytes of paroxysmal nocturnal hemoglobinuria to hemolysis in acidified serum. Blood. 1991;78: 820–9.
14. Harder MJ, Kuhn N, Schrezenmeier H, Höchsmann B, von Zabern I, Weinstock C, et al. Incomplete inhibition by eculizumab: mechanistic evidence for residual C5 activity during strong complement activation. Blood. 2017;129: 970–980.
15. Choi CW, Jang JH, Kim JS, Jo D-Y, Lee J-H, Kim S-H, et al. Efficacy of eculizumab in paroxysmal nocturnal hemoglobinuria patients with or without aplastic anemia: prospective study of a Korean PNH cohort. Blood Res. 2017;52: 207–211.
