# Supplementary material for: Modeling the activation of the alternative complement pathway and its effects on hemolysis in health and disease
Source: PLoS Comput Biol. 2020 Oct 2;16(10):e1008139. doi: 10.1371/journal.pcbi.1008139 (PMC7531836; doi:10.1371/journal.pcbi.1008139)
Supplement: S2 Table — Initial conditions of the differential equations describing the change in protein concentrations over time. For in vivo simulations, these initial values coincide with the steady state levels. For unlisted proteins and complexes, the initial concentration was assumed equal to 0 or to their steady state level when simulating the in vitro or in vivo setting, respectively. (PDF) [file pcbi.1008139.s006.pdf]

**Complement protein**	**Initial concentration (uM)**
C3	5.40
C5	0.37
C6	0.50
C7	0.50
C8	0.36
C9	0.90
FB	2.20
FD	0.083
FI	0.40
P	0.47
FH	3.20
CR1	0.0083
DAF	0.027
Vn	6.00
Cn	0.42
CD59	0.21
Surface (concentration of C3b binding sites for a cell concentration of $5*10^{12}$ cells L^{-1})	12.1
