# Supplementary material for: Modeling the activation of the alternative complement pathway and its effects on hemolysis in health and disease
Source: PLoS Comput Biol. 2020 Oct 2;16(10):e1008139. doi: 10.1371/journal.pcbi.1008139 (PMC7531836; doi:10.1371/journal.pcbi.1008139)
Supplement: S3 Table — Cell species, publication, initial cell concentration, readout time and serum concentration for each of the simulated hemolytic experiments. (PDF) [file pcbi.1008139.s007.pdf]

Cell species	Publication	Initial cell concentration (cells L^{-1})		Readout time (min)		Serum concentration (%)	
		Experim ent	Model	Experiment	Model	Experime nt	Model
Rabbit	[1]	$2.0*10^{11}$	$2.0*10^{11}$	20	20	0–50	0–50
Rabbit	[2]	$8.3*10^{10}$	$8.3*10^{10}$	30	30	8.3	8.3
Rabbit	[3]	n/a	$1*10^{11}$ (assumed based on [2,4])	Variable (5–120, dependent on experiment). 120 minutes assumed for C3 and FD mixing experiments based on other experiments in the publication		20	20
Human	[5]	$2.08*10^{11}$	$2.08*10^{11}$	20	30	40	40
Human	[6]	n/a	$1*10^{11}$ (assumed based on [2,4])	n/a	30	50	50
Human	[7]	$1.25*10^{11}$	$1.25*10^{11}$	30	30	45	45
Human (PNH)	[8]	n/a	$0.75*10^{11}$ (assumed based on protocol and common stock solution concentration of erythrocytes)	1440	1440	75	75

References

1. Pangburn MK. Cutting edge: localization of the host recognition functions of complement factor H at the carboxyl-terminal: implications for hemolytic uremic syndrome. J Immunol. 2002;169: 4702–6.
2. Thanassi JA, Dharaben P, Yang G, Galvan M, Yhao Y, Fabrycki J, et al. Comparison of complement functional assays: Differential sensitivities of hemolysis and Wieslab assays to levels of complement proteins C5, factor B, and factor D. XXVI ICW Kanazawa. 2016. pp. 1035–1226.
3. Wu X, Hutson I, Akk AM, Mascharak S, Pham CTN, Hourcade DE, et al. Contribution of Adipose-Derived Factor D/Adipsin to Complement Alternative Pathway Activation: Lessons from Lipodystrophy. J Immunol. 2018;200: 2786–2797.
4. Pangburn MK, Rawal N. Structure and function of complement C5 convertase enzymes. Biochem Soc Trans. 2002;30: 1006–10.
5. Ferreira VP, Pangburn MK. Factor H mediated cell surface protection from complement is

critical for the survival of PNH erythrocytes. Blood. 2007;110: 2190–2.
6. Lesher AM, Zhou L, Kimura Y, Sato S, Gullipalli D, Herbert AP, et al. Combination of factor H mutation and properdin deficiency causes severe C3 glomerulonephritis. J Am Soc Nephrol. 2013;24: 53–65.
7. Wilcox LA, Ezzell JL, Bernshaw NJ, Parker CJ. Molecular basis of the enhanced susceptibility of the erythrocytes of paroxysmal nocturnal hemoglobinuria to hemolysis in acidified serum. Blood. 1991;78: 820–9.
8. Harder MJ, Kuhn N, Schrezenmeier H, Höchsmann B, von Zabern I, Weinstock C, et al. Incomplete inhibition by eculizumab: mechanistic evidence for residual C5 activity during strong complement activation. Blood. 2017;129: 970–980.
