# Supplementary material for: Modeling the activation of the alternative complement pathway and its effects on hemolysis in health and disease
Source: PLoS Comput Biol. 2020 Oct 2;16(10):e1008139. doi: 10.1371/journal.pcbi.1008139 (PMC7531836; doi:10.1371/journal.pcbi.1008139)
Supplement: S4 Table — Estimation of the systemic half-life of complement proteins based on their molecular weight as described in S4 Appendix. (PDF) [file pcbi.1008139.s008.pdf]

Complement protein	MW (kDa)	Half-life (h)	Source of half-life value	Synthesis rate (mg kg^{-1} day^{-1})		FCR (% h^{-1})[b]		Source of synthesis rate and FCR values
				Experiment	Model[a]	Experiment	Model	
C3	185	41.7	Calculated from MW	14.4–26.6<br>13.2<br>10.3–27.9	30.1	1.1–1.8<br>1.6<br>1.3–1.8	2.9	[1–5]
C5	190	43.0	Calculated from MW	1.7–3.2	1.7	1.5–2.1	1.6	[3,6]
C6	105	20.2	Calculated from MW	n/a	2.1	n/a	3.4	
C7	92	16.8	Calculated from MW	n/a	2.3	n/a	4.1	
C8	151	32.8	Calculated from MW	n/a	1.2	n/a	2.1	
C9	71	11.6	Calculated from MW	1.68	3.9	2.9	6.0	[7]
FD	24	2.95	Calculated from MW	0.93–1.72	0.5	50–74.1	23.1	[8]
FB	93	17.0	Calculated from MW	3.29<br>3.36–5.04	31.0	1.6<br>1.7–2.2	14.7	[3,4,9]
FI	88	15.7	Calculated from MW	n/a	2.241.6	n/a	4.4	
P	53	7.6	Calculated from MW	0.1–0.34	2.3	0.78–1.08	9.1	[10]
FH	155	33.8	Calculated from MW	n/a	10.5	1.32	2.1	[7,11]
Vn	75	12.5	Calculated from MW	n/a	28.4	n/a	5.5	
Cn	75	12.5	Calculated from MW	n/a	2.0	n/a	5.5	
iC3b	176	39.4	Calculated from MW					
Ba	33	4.1	Calculated from MW					
Bb	60	9.1	Calculated from MW					

C3a	9.1	0.50	[12,13]					
C5a	10.4	0.017	[14,15]					
C3dg	38.9	0.067	[12,16]					

[a] For a comparison to literature values, a body weight of 70 kg and a plasma volume of 3 L was assumed to convert the estimated synthesis rate from M s^{-1} to mg kg^{-1} day^{-1}.
[b] Calculated from plasma levels in S2 Table.

References

1. Swaak AJG, Hannema A, Vogelaar C, Boom FA, van Es L, van Aalst R, et al. Determination of the half-life of C3 in patients and its relation to the presence of C3-breakdown products and/or circulating immune complexes. Rheumatol Int. 1982;2: 161–6.
2. Charlesworth JA, Peake PW, Golding J, Mackie JD, Pussell BA, Timmermans V, et al. Hypercatabolism of C3 and C4 in active and inactive systemic lupus erythematosus. Ann Rheum Dis. 1989;48: 153–9.
3. Sissons JGP, Liebowitch J, Amos N, Peters DK. Metabolism of the fifth component of complement, and its relation to metabolism of the third component, in patients with complement activation. J Clin Invest. 1977;59: 704–15.
4. Charlesworth JA, Williams DG, Sherington E, Peters DK. Metabolism of the third component of complement (C3) in normal human subjects. Clin Sci Mol Med. 1974;46: 223–9.
5. Wilson WA, Thomas EJ, Sissons JG. Complement activation in asymptomatic patients with sickle cell anaemia. Clin Exp Immunol. 1979;36: 130–9.
6. Hepburn NJ, Williams AS, Nunn MA, Chamberlain-Banoub JC, Hamer J, Morgan BP, et al. In vivo characterization and therapeutic efficacy of a C5-specific inhibitor from the soft tick Ornithodoros moubata. J Biol Chem. 2007;282: 8292–9.
7. Greenstein JD, Peake PW, Charlesworth JA. The metabolism of C9 in normal subjects and in patients with autoimmune disease. Clin Exp Immunol. 1996;104: 160–6.
8. Pascual M, Steiger G, Estreicher J, Macon K, Volanakis JE, Schifferli JA. Metabolism of complement factor D in renal failure. Kidney Int. 1988;34: 529–36.
9. Kaplan RA, Curd JG, Deheer DH, Carson DA, Pangburn MK, Müller-Eberhard HJ, et al. Metabolism of C4 and factor B in rheumatoid arthritis. Relation to rheumatoid factor. Arthritis Rheum. 1980;23: 911–20.
10. Ziegler JB, Rosen FS, Alper CA, Grupe W, Lepow IH. Metabolism of properdin in normal subjects and patients with renal disease. J Clin Invest. 1975;56: 761–7.
11. Charlesworth JA, Scott DM, Pussell BA, Peters DK. Metabolism of human beta 1H: studies in man and experimental animals. Clin Exp Immunol. 1979;38: 397–404.
12. Nilsson B, Ekdahl KN. Complement diagnostics: concepts, indications, and practical guidelines. Clin Dev Immunol. 2012;2012: 962702.
13. Norda R, Schött U, Berséus O, Åkerblom O, Nilsson B, Ekdahl KN, et al. Complement activation products in liquid stored plasma and C3a kinetics after transfusion of autologous plasma. Vox Sang. 2012;102: 125–133.
14. Oppermann M, Götze O. Plasma clearance of the human C5a anaphylatoxin by binding to

leucocyte C5a receptors. Immunology. 1994;82: 516.
15. Kirschfink M, Mollnes TE. Modern complement analysis. Clin Diagn Lab Immunol. 2003;10: 982–9.
16. Teisner B, Brandslund I, Grunnet N, Hansen LK, Thellesen J, Svehag SE. Acute complement activation during an anaphylactoid reaction to blood transfusion and the disappearance rate of C3c and C3d from the circulation. J Clin Lab Immunol. 1983;12: 63–67.
