# Supplementary material for: Modeling the activation of the alternative complement pathway and its effects on hemolysis in health and disease
Source: PLoS Comput Biol. 2020 Oct 2;16(10):e1008139. doi: 10.1371/journal.pcbi.1008139 (PMC7531836; doi:10.1371/journal.pcbi.1008139)
Supplement: S1 Appendix — List of biochemical reactions of the AP, complement protein turnover reactions and reactions involving eculizumab. (PDF) [file pcbi.1008139.s009.pdf]

Overview

Reaction no.	Reactions
1 – 111	Biochemical reactions of the AP
112 – 129	Complement protein turnover reactions (production)
130 – 223	Complement protein turnover reactions (degradation)
224 – 226	Eculizumab reactions

Biochemical reactions of the AP.

Reaction no.	Reaction	Reaction rate	Reaction location
1	C3 -> [C3(H2O) fluid]	C3 * [k_p_C3(H20)]	Fluid phase
2	[C3(H2O) fluid] + B <-> [C3(H2O)B fluid]	[k_p_C3(H20)B] * [C3(H2O) fluid] * B - [k_m_C3(H20)B] * [C3(H2O)B fluid]	Fluid phase
3	[C3(H2O) fluid] + H <-> [C3(H2O)H fluid]	[k_p_C3bH] * [C3(H2O) fluid] * H - [k_m_C3bH] * [C3(H2O)H fluid]	Fluid phase
4	[C3(H2O) fluid] + CR1 <-> [C3(H2O)CR1 fluid]	[k_p_C3bCR1] * [C3(H2O) fluid] * CR1 - [k_m_C3bCR1] * [C3(H2O)CR1 fluid]	Fluid phase
5	[C3(H2O)Bb fluid] -> [C3(H2O) fluid] + Bb	[k_m_C3(H2O)Bb] * [C3(H2O)Bb fluid]	Fluid phase
6	[C3(H2O)BbH fluid] -> H + Bb + [C3(H2O) fluid]	[k_m_C3bBbH decay] * [C3(H2O)BbH fluid]	Fluid phase
7	[C3(H2O)B fluid] + D -> [C3(H2O)Bb fluid] + Ba + D	[k_D_cat_C3(H2O)B] * D * [C3(H2O)B fluid] / ([K_D_m_C3(H2O)B] + [C3bB fluid] + [C3(H2O)B fluid] + [C3bB host])	Fluid phase
8	[C3(H2O)Bb fluid] + H -> [C3(H2O)BbH fluid]	[k_p_C3bH] * [C3(H2O)Bb fluid] * H	Fluid phase
9	nfC3b + H2O -> [C3b fluid]	k_p_fC3b * nfC3b	Fluid phase

10	nhC3b + H2O -> [C3b fluid]	k_p_fC3b * nhC3b	Fluid phase
11	[C3b fluid] + B <-> [C3bB fluid]	k_p_C3bB * [C3b fluid] * B - k_m_C3bB * [C3bB fluid]	Fluid phase
12	[C3b fluid] + H <-> [C3bH fluid]	k_p_C3bH * [C3b fluid] * H - k_m_C3bH * [C3bH fluid]	Fluid phase
13	[C3b fluid] + CR1 <-> [C3bCR1 fluid]	k_p_C3bCR1 * [C3b fluid] * CR1 - k_m_C3bCR1 * [C3bCR1 fluid]	Fluid phase
14	[C3bBb fluid] -> Bb + [C3b fluid]	k_m_C3bBb * [C3bBb fluid]	Fluid phase
15	[C3bBbH fluid] -> H + Bb + [C3b fluid]	[k_m_C3bBbH decay] * [C3bBbH fluid]	Fluid phase
16	[C3bBbCR1 fluid] -> CR1 + Bb + [C3b fluid]	[k_m_C3bBbCR1 decay] * [C3bBbCR1 fluid]	Fluid phase
17	[C3bB fluid] + D -> [C3bBb fluid] + Ba + D	[k_D_cat_C3bB] * D * [C3bB fluid] / ([K_D_m_C3bB] + [C3bB fluid] + [C3(H2O)B fluid] + [C3bB host])	Fluid phase
18	[C3bBb fluid] + H -> [C3bBbH fluid]	[k_p_C3bH] * H * [C3bBb fluid]	Fluid phase
19	CR1 + [C3bBb fluid] -> [C3bBbCR1 fluid]	[k_p_C3bCR1] * CR1 * [C3bBb fluid]	Fluid phase
20	C3 -> C3a + nfC3b	[k_C3_cat_C3(H2O)Bb] * [C3] * [C3(H2O)Bb fluid] / ([K_C3_m_C3(H2O)Bb] + [C3])	Fluid phase
21	C3 + [C3bBb fluid] -> C3a + nfC3b + [C3bBb fluid]	[k_C3_cat_C3bBb] * [C3] * [C3bBb fluid] / ([K_C3_m_C3bBb] + [C3])	Fluid phase
22	[C3bH fluid] + I -> H + [iC3b fluid] + I	[k_FI_cat_C3bH] * I * [C3bH fluid] / ([K_FI_m_C3bH] + [C3bH fluid] + [C3bCR1 fluid] + [iC3bCR1 fluid] + [C3bH host] + [C3bCR1 host] + [iC3bCR1 host])	Fluid phase
23	[iC3b fluid] + CR1 <-> [iC3bCR1 fluid]	[k_p_iC3bCR1] * [iC3b fluid] * CR1 - [k_m_iC3bCR1] * [iC3bCR1 fluid]	Fluid phase

24	[C3bCR1 fluid] + I -> CR1 + [iC3b fluid] + I	[k_FI_cat_C3bH] * I * [C3bCR1 fluid] / ([K_FI_m_C3bH] + [C3bH fluid] + [C3bCR1 fluid] + [iC3bCR1 fluid] + [C3bH host] + [C3bCR1 host] + [iC3bCR1 host])	Fluid phase
25	[iC3bCR1 fluid] + I -> CR1 + [C3dg fluid] + I	[k_FI_cat_C3bH] * I * [iC3bCR1 fluid] / ([K_FI_m_C3bH] + [C3bH fluid] + [C3bCR1 fluid] + [iC3bCR1 fluid] + [C3bH host] + [C3bCR1 host] + [iC3bCR1 host])	Fluid phase
26	[hC5b7 fluid] -> [C5b7 micelle]	[k_p_C5b7 micelle] * [hC5b7 fluid]	Fluid phase
27	[hC5b7 fluid] + C8 <-> [C5b8 fluid]	[k_p_C5b8] * [hC5b7 fluid] * C8 - [k_m_C5b8] * [C5b8 fluid]	Fluid phase
28	[hC5b7 fluid] + Cn <-> [CnC5b7 fluid]	[k_p_CnC5b7] * [hC5b7 fluid] * Cn - [k_m_CnC5b7] * [CnC5b7 fluid]	Fluid phase
29	[hC5b7 fluid] + Vn <-> [VnC5b7 fluid]	[k_p_VnC5b7] * [hC5b7 fluid] * Vn - [k_m_VnC5b7] * [VnC5b7 fluid]	Fluid phase
30	[VnC5b7 fluid] + C8 <-> [VnC5b8 fluid]	[k_p_VnC5b8] * [VnC5b7 fluid] * C8 - [k_m_VnC5b8] * [VnC5b8 fluid]	Fluid phase
31	[VnC5b8 fluid] + C9 <-> [VnC5b9_1 fluid]	[k_p_VnC5b9] * [VnC5b8 fluid] * C9 - [k_m_VnC5b9] * [VnC5b9_1 fluid]	Fluid phase
32	[CnC5b7 fluid] + C8 <-> [CnC5b8 fluid]	[k_p_CnC5b8] * [CnC5b7 fluid] * C8 - [k_m_CnC5b8] * [CnC5b8 fluid]	Fluid phase
33	[CnC5b8 fluid] + C9 <-> [CnC5b9_1 fluid]	[k_p_CnC5b9] * [CnC5b8 fluid] * C9 - [k_m_CnC5b9] * [CnC5b9_1 fluid]	Fluid phase
34	[C5b8 fluid] + C9 <-> [C5b9_1 fluid]	[k_p_C5b9] * [C5b8 fluid] * C9 - [k_m_C5b9] * [C5b9_1 fluid]	Fluid phase
35	[C3bBbH fluid] -> [C3bBb fluid] + H	[k_m_C3bH] * [C3bBbH fluid]	Fluid phase
36	[C3bBbCR1 fluid] -> CR1 + [C3bBb fluid]	[k_m_C3bCR1] * [C3bBbCR1 fluid]	Fluid phase
37	[C3b fluid] + [C3bBb host] <-> [C3bBbC3b host]	k_p_C3bBbC3b * [C3b fluid] * [C3bBb host] - k_m_C3bBbC3b * [C3bBbC3b host]	Erythrocyte membrane

38	[C3bBbC3bCR1 host] -> CR1 + [C3b fluid] + [C3b host] + Bb	[k_m_C3bBbCR1 decay] * [C3bBbC3bCR1 host]	Erythrocyte membrane
39	[C3bBbC3bDAF host] -> DAF + [C3b fluid] + [C3b host] + Bb	[k_m_C3bBbDAF decay] * [C3bBbC3bDAF host]	Erythrocyte membrane
40	[C3bBbC3bH host] -> H + [C3b fluid] + [C3b host] + Bb	[k_m_C3bBbH decay] * [C3bBbC3bH host]	Erythrocyte membrane
41	nfC3b + [Surface host] -> [C3b host]	[k_p_C3b_surface] * [nfC3b] * [Surface host]	Erythrocyte membrane
42	[C3bBbP host] -> P + [C3b host] + Bb	[k_m_C3bBbP] * [C3bBbP host]	Erythrocyte membrane
43	C3 + [C3bBbP host] -> C3a + nhC3b + [C3bBbP host]	[k_C3_cat_C3bBbP] * [C3] * [C3bBbP host] / ([K_C3_m_C3bBbP] + [C3])	Erythrocyte membrane
44	[C3bBbP host] + nhC3b -> [C3bBbC3bP host]	[k_p_C3bBbC3b] * [C3bBbP host] * nhC3b	Erythrocyte membrane
45	[C3bBP host] + D -> [C3bBbP host] + Ba + D	[k_D_cat_C3bB] * D * [C3bBP host] / ([K_D_m_C3(H2O)B] + [C3bB fluid] + [C3(H2O)B fluid] + [C3bB host]+ [C3bBP host])	Erythrocyte membrane
46	[C3bBb host] + P -> [C3bBbP host]	[k_p_C3bP] * [C3bBb host] * [P]	Erythrocyte membrane
47	[C3bBbP host] + [C3b fluid] -> [C3bBbC3bP host]	[k_p_C3bBbC3b] * [C3bBbP host] * [C3b fluid]	Erythrocyte membrane
48	[iC3b host] + P <-> [iC3bP host]	[k_p_iC3bP] * [iC3b host] * [P] - [k_m_iC3bP] * [iC3bP host]	Erythrocyte membrane
49	[C3bBbC3b host] + P <-> [C3bBbC3bP host]	[k_p_C3bP] * [C3bBbC3b host] * P - [k_m_C3bP] * [C3bBbC3bP host]	Erythrocyte membrane
50	[C3bBbC3bPC5 host] -> [C3bBbC3bC5 host] + P	[k_m_C3bP] * [C3bBbC3bPC5 host]	Erythrocyte membrane
51	[C3bBbC3bPC5b host] -> [C3bBbC3bC5b host] + P	[k_m_C3bP] * [C3bBbC3bPC5b host]	Erythrocyte membrane

52	[C3bBbC3bPC5bC6 host] -> [C3bBbC3bC5bC6 host] + P	[k_m_C3bP] * [C3bBbC3bPC5bC6 host]	Erythrocyte membrane
53	[C3bBbC3bPC5bC6 host] + C7 -> [C3bBbC3bP host] + [hC5b7 fluid]	[k_p_C5b7] * [C3bBbC3bPC5bC6 host] * C7	Erythrocyte membrane
54	[C3bBbC3bPC5b host] -> [C3bBbC3bP host] + C5b	[k_m_C5b] * [C3bBbC3bPC5b host]	Erythrocyte membrane
55	[C3bBbC3bP host] + C5 -> C5a + [C3bBbC3bPC5b host]	[k_C5_cat_C3bBbC3b] * [C3bBbC3bP host] * C5 / ([K_C5_m_C3bBbC3b] + C5)	Erythrocyte membrane
56	[C3bBbC3bPC5b host] + C6 <-> [C3bBbC3bPC5bC6 host]	[k_p_C3bBbC3bC5bC6] * [C3bBbC3bPC5b host] * C6 - [k_m_C3bBbC3bC5bC6] * [C3bBbC3bPC5bC6 host]	Erythrocyte membrane
57	C3 + [C3bBb host] -> C3a + nhC3b + [C3bBb host]	[k_C3_cat_C3bBb] * [C3] * [C3bBb host] / ([K_C3_m_C3bBb] + [C3])	Erythrocyte membrane
58	nhC3b + [Surface host] -> [C3b host]	[k_p_hC3b] * [nhC3b] * [Surface host] * 15.6	Erythrocyte membrane
59	nhC3b + [C3bBb host] -> [C3bBbC3b host]	[k_p_C3bBbC3b] * [nhC3b] * [C3bBb host]	Erythrocyte membrane
60	[hC5b7 fluid] + [Surface host] + [Surface host] + [Surface host] + [Surface host] -> [C5b7 host]	[k_p_C5b7 surface] * [hC5b7 fluid] * [Surface host] * 1.4	Erythrocyte membrane
61	[C3b host] + CR1 <-> [C3bCR1 host]	[k_p_C3bCR1] * [C3b host] * CR1 - [k_m_C3bCR1] * [C3bCR1 host]	Erythrocyte membrane
62	[C3b host] + H <-> [C3bH host]	[k_p_C3bH_surf] * [C3b host] * H - [k_m_C3bH] * [C3bH host]	Erythrocyte membrane
63	[C3b host] + B <-> [C3bB host]	[k_p_C3bB] * [C3b host] * B - [k_m_C3bB] * [C3bB host]	Erythrocyte membrane
64	[C3bBbH host] -> H + Bb + [C3b host]	[k_m_C3bBbH decay] * [C3bBbH host]	Erythrocyte membrane
65	[C3bBb host] -> Bb + [C3b host]	[k_m_C3bBb] * [C3bBb host]	Erythrocyte membrane

66	[C3bBbCR1 host] -> CR1 + Bb + [C3b host]	[k_m_C3bBbCR1 decay] * [C3bBbCR1 host]	Erythrocyte membrane
67	[C3bBbDAF host] -> DAF + Bb + [C3b host]	[k_m_C3bBbDAF decay] * [C3bBbDAF host]	Erythrocyte membrane
68	[C3bBbC3b host] -> Bb + [C3b host] + [C3b fluid]	[k_m_C3bBbC3b] * [C3bBbC3b host]	Erythrocyte membrane
69	[C3bB host] + D -> [C3bBb host] + Ba + D	[k_D_cat_C3bB] * D * [C3bB host] / ([K_D_m_C3(H2O)B] + [C3bB fluid] + [C3(H2O)B fluid] + [C3bB host]+ [C3bBP host])	Erythrocyte membrane
70	[C3bBb host] + H -> [C3bBbH host]	[k_p_C3bH_surf] * [C3bBb host] * H	Erythrocyte membrane
71	[C3bBb host] + CR1 -> [C3bBbCR1 host]	[k_p_C3bCR1] * [C3bBb host] * CR1	Erythrocyte membrane
72	[C3bBb host] + DAF -> [C3bBbDAF host]	[k_p_C3bBbDAF] * [C3bBb host] * DAF	Erythrocyte membrane
73	[C3bCR1 host] + I -> CR1 + [iC3b host] + I	[k_FI_cat_C3bH] * I * [C3bCR1 host] / ([K_FI_m_C3bH] + [C3bH fluid] + [C3bCR1 fluid] + [iC3bCR1 fluid] + [C3bH host] + [C3bCR1 host] + [iC3bCR1 host])	Erythrocyte membrane
74	[iC3b host] + CR1 <-> [iC3bCR1 host]	[k_p_iC3bCR1] * [iC3b host] * CR1 - [k_m_iC3bCR1] * [iC3bCR1 host]	Erythrocyte membrane
75	[C3bH host] + I -> H + [iC3b host] + I	[k_FI_cat_C3bH] * I * [C3bH host] / ([K_FI_m_C3bH] + [C3bH fluid] + [C3bCR1 fluid] + [iC3bCR1 fluid] + [C3bH host] + [C3bCR1 host] + [iC3bCR1 host])	Erythrocyte membrane
76	[iC3bCR1 host] + I -> CR1 + [C3dg host] + I	[k_FI_cat_C3bH] * I * [iC3bCR1 host] / ([K_FI_m_C3bH] + [C3bH fluid] + [C3bCR1 fluid] + [iC3bCR1 fluid] + [C3bH host] + [C3bCR1 host] + [iC3bCR1 host])	Erythrocyte membrane
77	[C3bBbC3b host] + CR1 -> [C3bBbC3bCR1 host]	[k_p_C3bCR1] * [C3bBbC3b host] * CR1	Erythrocyte membrane
78	[C3bBbC3b host] + DAF -> [C3bBbC3bDAF host]	[k_p_C3bBbDAF] * [C3bBbC3b host] * DAF	Erythrocyte membrane

79	[C3bBbC3b host] + H -> [C3bBbC3bH host]	[k_p_C3bH_surf] * [C3bBbC3b host] * H	Erythrocyte membrane
80	[C3bBbC3bC5b host] -> C5b + [C3bBbC3b host]	[k_m_C5b] * [C3bBbC3bC5b host]	Erythrocyte membrane
81	[C3bBbC3bC5bC6 host] + C7 -> [C3bBbC3b host] + [hC5b7 fluid]	[k_p_C5b7] * [C3bBbC3bC5bC6 host] * C7	Erythrocyte membrane
82	[C3bBbC3b host] + C5 -> C5a + [C3bBbC3bC5b host]	[k_C5_cat_C3bBbC3b] * [C3bBbC3b host] * C5 / ([K_C5_m_C3bBbC3b] + C5)	Erythrocyte membrane
83	[C3bBbC3bC5b host] + C6 <-> [C3bBbC3bC5bC6 host]	[k_p_C3bBbC3bC5bC6] * [C3bBbC3bC5b host] * C6 - [k_m_C3bBbC3bC5bC6] * [C3bBbC3bC5bC6 host]	Erythrocyte membrane
84	[C5b7 host] + C8 -> [C5b8 host]	[k_p_C5b8] * [C5b7 host] * C8	Erythrocyte membrane
85	[C5b8 host] + C9 -> [C5b9_1 host]	[k_p_C5b9] * [C5b8 host] * C9	Erythrocyte membrane
86	[C5b9_1 host] + C9 -> [C5b9_2 host]	[k_p_C5b9] * [C5b9_1 host] * C9	Erythrocyte membrane
87	[C5b9_2 host] + C9 -> [C5b9_3 host]	[k_p_C5b9] * [C5b9_2 host] * C9	Erythrocyte membrane
88	[C5b9_3 host] + C9 -> [C5b9_4 host]	[k_p_C5b9] * [C5b9_3 host] * C9	Erythrocyte membrane
89	[C5b9_4 host] + C9 -> [C5b9_5 host]	[k_p_C5b9] * [C5b9_4 host] * C9	Erythrocyte membrane
90	[C5b9_5 host] + C9 -> [C5b9_6 host]	[k_p_C5b9] * [C5b9_5 host] * C9	Erythrocyte membrane
91	[C5b9_6 host] + C9 -> [C5b9_7 host]	[k_p_C5b9] * [C5b9_6 host] * C9	Erythrocyte membrane
92	[C5b9_7 host] + C9 -> [C5b9_8 host]	[k_p_C5b9] * [C5b9_7 host] * C9	Erythrocyte membrane

93	[C5b9_8 host] + C9 -> [C5b9_9 host]	[k_p_C5b9] * [C5b9_8 host] * C9	Erythrocyte membrane
94	[C5b9_9 host] + C9 -> [C5b9_10 host]	[k_p_C5b9] * [C5b9_9 host] * C9	Erythrocyte membrane
95	[C5b9_10 host] + C9 -> [C5b9_11 host]	[k_p_C5b9] * [C5b9_10 host] * C9	Erythrocyte membrane
96	[C5b9_11 host] + C9 -> [C5b9_12 host]	[k_p_C5b9] * [C5b9_11 host] * C9	Erythrocyte membrane
97	[C5b9_12 host] + C9 -> [C5b9_13 host]	[k_p_C5b9] * [C5b9_12 host] * C9	Erythrocyte membrane
98	[C5b9_13 host] + C9 -> [C5b9_14 host]	[k_p_C5b9] * [C5b9_13 host] * C9	Erythrocyte membrane
99	[C5b9_14 host] + C9 -> [C5b9_15 host]	[k_p_C5b9] * [C5b9_14 host] * C9	Erythrocyte membrane
100	[C5b9_15 host] + C9 -> [C5b9_16 host]	[k_p_C5b9] * [C5b9_15 host] * C9	Erythrocyte membrane
101	[C5b9_16 host] + C9 -> [C5b9_17 host]	[k_p_C5b9] * [C5b9_16 host] * C9	Erythrocyte membrane
102	[C5b9_17 host] + C9 -> [MAC host]	[k_p_C5b9] * [C5b9_17 host] * C9	Erythrocyte membrane
103	[C5b9_1 host] + CD59 <-> [CD59C5b9_1 host]	[k_p_CD59C5b9] * [C5b9_1 host] * CD59 - [k_m_CD59C5b9] * [CD59C5b9_1 host]	Erythrocyte membrane
104	[C3bBbH host] -> [C3bBb host] + H	[k_m_C3bH] * [C3bBbH host]	Erythrocyte membrane
105	[C3bBbC3bH host] -> [C3bBbC3b host] + H	[k_m_C3bH] * [C3bBbC3bH host]	Erythrocyte membrane
106	[C3bBbCR1 host] -> [C3bBb host] + CR1	[k_m_C3bCR1] * [C3bBbCR1 host]	Erythrocyte membrane
107	[C3bBbC3bCR1 host] -> [C3bBbC3b host] + CR1	[k_m_C3bCR1] * [C3bBbC3bCR1 host]	Erythrocyte membrane

108	[C3bBbDAF host] -> [C3bBb host] + DAF	[k_m_C3bBbDAF] * [C3bBbDAF host]	Erythrocyte membrane
109	[C3bBbC3bDAF host] -> [C3bBbC3b host] + DAF	[k_m_C3bBbDAF] * [C3bBbC3bDAF host]	Erythrocyte membrane
110	[C3bBbP host] -> [C3bBb host] + P	[k_m_C3bP] * [C3bBbP host]	Erythrocyte membrane
111	[C3bBbC3bP host] -> Bb + [C3b host] + [C3b fluid] + P	[k_m_C3bBbC3bP] * [C3bBbC3bP host]	Erythrocyte membrane

Complement protein turnover reactions.

Reaction no.	Reaction	Reaction rate
112	null -> [Surface host]	k_pr_surface
113	[Surface host] -> null	[Surface host] * k_el_S
114	null -> C3	k_pr_C3
115	null -> C5	k_pr_C5
116	null -> C6	k_pr_C6
117	null -> C7	k_pr_C7
118	null -> C8	k_pr_C8
119	null -> C9	k_pr_C9
120	null -> B	k_pr_B
121	null -> D	k_pr_D
122	null -> P	k_pr_P
123	null -> I	k_pr_I
124	null -> H	k_pr_H
125	null -> Vn	k_pr_Vn
126	null -> Cn	k_pr_Cn
127	null -> CR1	k_pr_CR1
128	null -> DAF	k_pr_DAF
129	null -> CD59	k_pr_CD59

130	C3 -> null	C3 * k_el_C3
131	C5 -> null	C5 * k_el_C5
132	C6 -> null	C6 * k_el_C6
133	C7 -> null	C7 * k_el_C7
134	C8 -> null	C8 * k_el_C8
135	C9 -> null	C9 * k_el_C9
136	B -> null	B * k_el_B
137	D -> null	D * k_el_D
138	P -> null	P * k_el_P
139	I -> null	I * k_el_I
140	H -> null	H * k_el_H
141	Vn -> null	Vn * k_el_Vn
142	Cn -> null	Cn * k_el_Cn
143	CR1 -> null	CR1 * k_el_S
144	CD59 -> null	CD59 * k_el_S
145	DAF -> null	DAF * k_el_S
146	[C3(H2O) fluid] -> null	[C3(H2O) fluid] * k_el_C3
147	[C3(H2O)B fluid] -> null	[C3(H2O)B fluid] * k_el_C3
148	[C3(H2O)H fluid] -> null	[C3(H2O)H fluid] * k_el_C3
149	[C3(H2O)CR1 fluid] -> null	[C3(H2O)CR1 fluid] * k_el_S

150	[C3(H2O)Bb fluid] -> null	[C3(H2O)Bb fluid] * k_el_C3
151	[C3(H2O)BbH fluid] -> null	[C3(H2O)BbH fluid] * k_el_C3
152	[C3b fluid] -> null	[C3b fluid] * k_el_C3
153	[C3bB fluid] -> null	[C3bB fluid] * k_el_C3
154	[C3bH fluid] -> null	[C3bH fluid] * k_el_C3
155	[C3bCR1 fluid] -> null	[C3bCR1 fluid] * k_el_S
156	[C3bBb fluid] -> null	[C3bBb fluid] * k_el_C3
157	[C3bBbH fluid] -> null	[C3bBbH fluid] * k_el_C3
158	[C3bBbCR1 fluid] -> null	[C3bBbCR1 fluid] * k_el_S
159	nfC3b -> null	nfC3b * k_el_C3
160	nhC3b -> null	nhC3b * k_el_C3
161	Ba -> null	Ba * k_el_Ba
162	Bb -> null	Bb * k_el_Bb
163	C3a -> null	C3a * k_el_C3a
164	C5a -> null	C5a * k_el_C5a
165	[hC5b7 fluid] -> null	[hC5b7 fluid] * k_el_C5
166	[iC3b fluid] -> null	[iC3b fluid] * k_el_iC3b
167	[iC3bCR1 fluid] -> null	[iC3bCR1 fluid] * k_el_S
168	[C3dg fluid] -> null	[C3dg fluid] * k_el_C3dg
169	[C5b7 micelle] -> null	[C5b7 micelle] * k_el_C5

170	[C5b8 fluid] -> null	[C5b8 fluid] * k_el_C5
171	[CnC5b7 fluid] -> null	[CnC5b7 fluid] * k_el_C5
172	[VnC5b7 fluid] -> null	[VnC5b7 fluid] * k_el_C5
173	[VnC5b8 fluid] -> null	[VnC5b8 fluid] * k_el_C5
174	[VnC5b9_1 fluid] -> null	[VnC5b9_1 fluid] * k_el_C5
175	[CnC5b8 fluid] -> null	[CnC5b8 fluid] * k_el_C5
176	[CnC5b9_1 fluid] -> null	[CnC5b9_1 fluid] * k_el_C5
177	[C5b9_1 fluid] -> null	[C5b9_1 fluid] * k_el_C5
178	[C3bBb host] -> [Surface host]	[C3bBb host] * k_el_S
179	[C3bBbC3b host] -> [Surface host]	[C3bBbC3b host] * k_el_S
180	[C3bBbC3bCR1 host] -> [Surface host]	[C3bBbC3bCR1 host] * k_el_S
181	[C3b host] -> [Surface host]	[C3b host] * k_el_S
182	[C3bBbC3bDAF host] -> [Surface host]	[C3bBbC3bDAF host] * k_el_S
183	[C3bBbC3bH host] -> [Surface host]	[C3bBbC3bH host] * k_el_S
184	[C3bBbP host] -> [Surface host]	[C3bBbP host] * k_el_S
185	[C3bBbC3bP host] -> [Surface host]	[C3bBbC3bP host] * k_el_S
186	[C3bBP host] -> [Surface host]	[C3bBP host] * k_el_S
187	[iC3b host] -> [Surface host]	[iC3b host] * k_el_S
188	[iC3bP host] -> [Surface host]	[iC3bP host] * k_el_S
189	[C3bBbC3bPC5 host] -> [Surface host]	[C3bBbC3bPC5 host] * k_el_S

190	[C3bBbC3bC5 host] -> [Surface host]	[C3bBbC3bC5 host] * k_el_S
191	[C3bBbC3bPC5b host] -> [Surface host]	[C3bBbC3bPC5b host] * k_el_S
192	[C3bBbC3bC5b host] -> [Surface host]	[C3bBbC3bC5b host] * k_el_S
193	[C3bBbC3bPC5bC6 host] -> [Surface host]	[C3bBbC3bPC5bC6 host] * k_el_S
194	[C3bBbC3bC5bC6 host] -> [Surface host]	[C3bBbC3bC5bC6 host] * k_el_S
195	[C5b7 host] -> [Surface host]	[C5b7 host] * k_el_S
196	[C3bCR1 host] -> [Surface host]	[C3bCR1 host] * k_el_S
197	[C3bH host] -> [Surface host]	[C3bH host] * k_el_S
198	[C3bB host] -> [Surface host]	[C3bB host] * k_el_S
199	[C3bBbH host] -> [Surface host]	[C3bBbH host] * k_el_S
200	[C3bBbCR1 host] -> [Surface host]	[C3bBbCR1 host] * k_el_S
201	[C3bBbDAF host] -> [Surface host]	[C3bBbDAF host] * k_el_S
202	[iC3bCR1 host] -> [Surface host]	[iC3bCR1 host] * k_el_S
203	[C3dg host] -> [Surface host]	[C3dg host] * k_el_S
204	[C5b8 host] -> [Surface host]	[C5b8 host] * k_el_S
205	[C5b9_1 host] -> [Surface host]	[C5b9_1 host] * k_el_S
206	[C5b9_2 host] -> [Surface host]	[C5b9_2 host] * k_el_S
207	[C5b9_3 host] -> [Surface host]	[C5b9_3 host] * k_el_S
208	[C5b9_4 host] -> [Surface host]	[C5b9_4 host] * k_el_S

209	[C5b9_5 host] -> [Surface host]	[C5b9_5 host] * k_el_S
210	[C5b9_6 host] -> [Surface host]	[C5b9_6 host] * k_el_S
211	[C5b9_7 host] -> [Surface host]	[C5b9_7 host] * k_el_S
212	[C5b9_8 host] -> [Surface host]	[C5b9_8 host] * k_el_S
213	[C5b9_9 host] -> [Surface host]	[C5b9_9 host] * k_el_S
214	[C5b9_10 host] -> [Surface host]	[C5b9_10 host] * k_el_S
215	[C5b9_11 host] -> [Surface host]	[C5b9_11 host] * k_el_S
216	[C5b9_12 host] -> [Surface host]	[C5b9_12 host] * k_el_S
217	[C5b9_13 host] -> [Surface host]	[C5b9_13 host] * k_el_S
218	[C5b9_14 host] -> [Surface host]	[C5b9_14 host] * k_el_S
219	[C5b9_15 host] -> [Surface host]	[C5b9_15 host] * k_el_S
220	[C5b9_16 host] -> [Surface host]	[C5b9_16 host] * k_el_S
221	[C5b9_17 host] -> [Surface host]	[C5b9_17 host] * k_el_S
222	[MAC] -> [Surface host]	[MAC host] * k_el_S
223	[CD59C5b9_1 host] -> [Surface host]	[CD59C5b9_1 host] * k_el_S

Reactions involving eculizumab.

Reaction no.	Reaction	Reaction rate
224	C5 + eculizumab <-> [eculizumab C5]	C5 * eculizumab * kon_ecu - [eculizumab C5] * koff_ecu
225	eculizumab -> null	eculizumab * k_el_ecu
226	[eculizumab C5] -> null	[eculizumab C5] * k_el_ecu
