# Supplementary material for: Modeling the activation of the alternative complement pathway and its effects on hemolysis in health and disease
Source: PLoS Comput Biol. 2020 Oct 2;16(10):e1008139. doi: 10.1371/journal.pcbi.1008139 (PMC7531836; doi:10.1371/journal.pcbi.1008139)
Supplement: S2 Appendix — List of model parameters. (PDF) [file pcbi.1008139.s010.pdf]

Parameter	Value	Units	Description	Source
V	3.00000e+00	L	Reaction volume	Serum volume of a human
C_e	5.00000e+12	cells L^{-1}	Concentration of erythrocytes	Physiological value
V_e	9.00000e-14	L	Mean corpuscular volume	Physiological value
N_h_e	2.70000e+08	$cell^{-1}$	Number of hemoglobin molecules per erythrocyte	Physiological value
MW_h	6.45000e+04	Da	Molecular weight of hemoglobin	Physiological value
Avogadro	6.02000e+23	mol^{-1}	Avogadro constant	Physical constant
LDHmax	1.49500e+03	U L^{-1}	Maximal LDH level	S1 Figure
H_LDH50	7.94000e+00	g dL^{-1}	Hemoglobin level at half-maximal LDH levels	S1 Figure
LDH0	2.96000e+02	U L^{-1}	Baseline LDH level	S1 Figure
MAC50	1.15000e+00	$cell^{-1}$	Number of MAC/cell resulting in 50% hemolysis	Fig. 2, S3 Appendix
k_p_C3(H20)	8.30000e-07	s^{-1}	Hydrolysis of C3(H2O)	[1]
k_p_C3(H20)B	2.13000e+05	M^{-1} s^{-1}	Association of Factor B to C3(H2O)	[2] (Estimation)
k_m_C3(H20)B	1.55000e-01	s^{-1}	Dissociation of complex C3(H2O)B	[2] (Estimation)
k_p_C3bH	1.10000e+06	M^{-1} s^{-1}	Association of Factor H to C3(H2O)	[3]
k_m_C3bH	5.90000e-02	s^{-1}	Dissociation of Factor H complexes	[3]
k_p_C3bH_surf	1.10000e+06	M^{-1} s^{-1}	Association of Factor H to surface bound proteins	[3]

k_p_C3bCR1	4.40000e+06	M^{-1} s^{-1}	Association of CR1 to C3b	[3]
k_m_C3bCR1	5.70000e-02	s^{-1}	Dissociation of CR1 complexes	[3]
k_m_C3(H2O)Bb	9.00000e-03	s^{-1}	Dissociation of complex C3(H2O)Bb	[5]
k_m_C3bBbH decay	7.70000e-02	s^{-1}	Decay of C3 convertase by Factor H	[2] (Assumption)
k_p_fC3b	1.20000e+04	s^{-1}	Association of nfC3b and nhC3b to water	[6]
k_p_C3bB	2.22590e+05	M^{-1} s^{-1}	Association of Factor B to C3b	Estimation
k_m_C3bB	1.55000e-01	s^{-1}	Dissociation of complex C3bB	[7]
k_m_C3bBb	7.70000e-03	s^{-1}	Dissociation of complex C3bBb	[5]
k_m_C3bBbCR1 decay	7.70000e-02	s^{-1}	Decay of C3 convertase by inhibitor CR1	[2] (Assumption)
k_p_C3bBbC3b	3.50000e+06	M^{-1} s^{-1}	Association of C3b to C3bBb	[8] (Optimization)
k_m_C3bBbC3b	3.80000e-03	s^{-1}	Dissociation of complex C3bBbC3b	[9]
k_m_C3bBbDAF decay	2.27560e-03	s^{-1}	Decay of C3 convertase by inhibitor DAF	Estimation
k_p_C3b_surfac e	2.16000e+09	M^{-1} s^{-1}	Attachment of nfC3b to erythrocyte	Estimation
k_p_hC3b	2.67000e+04	M^{-1} s^{-1}	Attachment of nhC3b to erythrocyte	[2] (Calculated)
k_p_C5b7 surface	4.20000e+08	M^{-1} s^{-1}	Attachment of C5b7 to erythrocyte	[2] (calculated)
k_p_C3bBbDAF	2.52950e+10	M^{-1} s^{-1}	Association of DAF to C3 convertase on erythrocyte	Estimation

k_p_iC3bCR1	2.00000e+03	$M^{-1} s^{-1}$	Association of iC3b to CR1	[2] (Estimation)
k_m_iC3bCR1	1.00000e-02	s^{-1}	Dissociation of complex iC3bCR1	[2] (Estimation)
k_m_C5b	3.80000e-02	s^{-1}	Dissociation of complex C3bBbC3bC5b	[10]
k_p_C5b7	7.30000e+05	$M^{-1} s^{-1}$	Association of C7 to C3bBbC3bC5bC6	[8,11,12]
k_m_C5b7	8.00000e-07	s^{-1}	Dissociation of complex C3bBbC3bC5bC6C7	[8,11,12]
k_p_C5b8	1.10000e+06	$M^{-1} s^{-1}$	Association of C8 to C5b7	[8,11,12]
k_m_C5b8	9.80000e-07	s^{-1}	Dissociation of complex C5b8	[8,11,12]
k_p_C5b9	2.80000e+06	$M^{-1} s^{-1}$	Association of C9 to C5b8	[8,11,12]
k_m_C5b9	1.00000e-06	s^{-1}	Dissociation of complex C5b9	[8,11,12]
k_p_CD59C5b9	6.03000e+11	$M^{-1} s^{-1}$	Association of CD59 to C5b9	Estimation
k_m_CD59C5b9	2.00000e-04	s^{-1}	Dissociation of complex CD59C5b9	[2] (Assumption)
k_p_C3bBbC3b C5bC6	7.74360e+04	$M^{-1} s^{-1}$	Association of C6 to C3bBbC3bC5b	Estimation
k_m_C3bBbC3b C5bC6	9.00000e-08	s^{-1}	Dissociation of complex C3bBbC3bC5bC6	[8,11]
k_p_C5b7 micelle	6.93000e+01	s^{-1}	Formation of C5b7 micelle in fluid	[12]
k_p_CnC5b7	4.10000e+05	$M^{-1} s^{-1}$	Association of Cn to C5b7	[2] (Estimation)
k_m_CnC5b7	4.00000e-03	s^{-1}	Dissociation of complex CnC5b7	[2] (Estimation)

k_p_VnC5b7	2.40000e+05	M^{-1} s^{-1}	Association of Vn to C5b7	[8,13]
k_m_VnC5b7	2.00000e-03	s^{-1}	Dissociation of complex VnC5b7	[2] (Assumption)
k_p_VnC5b8	1.10000e+06	M^{-1} s^{-1}	Association of C8 to VnC5b7	[8,11,12]
k_m_VnC5b8	9.80000e-07	s^{-1}	Dissociation of complex VnC5b8	[2] (Assumption)
k_p_VnC5b9	2.80000e+06	M^{-1} s^{-1}	Association of C9 to VnC5b8	[2] (Assumption)
k_m_VnC5b9	1.00000e-06	s^{-1}	Dissociation of complex VnC5b9	[2] (Assumption)
k_p_CnC5b8	1.10000e+06	M^{-1} s^{-1}	Association of Cn to C5b8	[2] (Assumption)
k_m_CnC5b8	7.30000e+05	s^{-1}	Dissociation of complex CnC5b8	[2] (Assumption)
k_p_CnC5b9	2.80000e+06	M^{-1} s^{-1}	Association of C9 to CnC5b8	[2] (Assumption)
k_m_CnC5b9	1.00000e-06	s^{-1}	Dissociation of complex CnC5b9	[2] (Assumption)
k_D_cat_C3(H2O)B	2.10000e+00	s^{-1}	Activation of complex C3(H2O)B by enzyme Factor D	[2] (Estimation)
K_D_m_C3(H2O)B	1.00000e-07	M	Activation of complex C3(H2O)B by enzyme Factor D	[2] (Estimation)
k_D_cat_C3bB	2.10000e+00	s^{-1}	Activation of complex C3bB by enzyme Factor D	[8] (Optimization)
K_D_m_C3bB	1.00000e-07	M	Activation of complex C3bB by enzyme Factor D	[8] (Optimization)
k_C3_cat_C3(H2O)Bb	1.80000e+00	s^{-1}	Cleavage of C3 by C3 convertase C3(H20)Bb	[2] (Estimation)
K_C3_m_C3(H2O)Bb	4.19000e-06	M	Cleavage of C3 by C3 convertase C3(H20)Bb	Estimation

k_C3_cat_C3bBb	1.80000e+00	s^{-1}	Cleavage of C3 by C3 convertase C3bBb	[5]
K_C3_m_C3bBb	5.90000e-06	M	Cleavage of C3 by C3 convertase C3bBb	[5]
k_FI_cat_C3bH	1.30000e+00	s^{-1}	Cleavage of C3b by inhibitor Factor I	[4]
K_FI_m_C3bH	2.50000e-07	M	Cleavage of C3b by inhibitor Factor I	[4]
k_C5_cat_C3bBbC3b	3.00000e-03	s^{-1}	Cleavage of C5 by C5 convertase C3bBbC3b	[3,14–16]
K_C5_m_C3bBbC3b	1.60000e-08	M	Cleavage of C5 by C5 convertase C3bBbC3b	[3,14–16]
k_p_C3bP	1.23700e+08	$M^{-1} s^{-1}$	Association of P to C3b on cell	Estimation
k_m_C3bP	5.00000e-04	s^{-1}	Dissociation of Properdin complexes	[17]
k_p_C3bBP	2.13000e+05	$M^{-1} s^{-1}$	Association of Factor B to C3bP	[8]
k_m_C3bBbP	7.70000e-04	s^{-1}	Dissociation of complex C3bBbP	[17]
k_p_iC3bP	3.00000e+06	$M^{-1} s^{-1}$	Association of Properdin to iC3b	[8] (Optimization)
k_m_iC3bP	3.80000e-04	s^{-1}	Dissociation of Properdin from iC3b	[17]
k_C3_cat_C3bBbP	3.10000e+00	s^{-1}	Cleavage of C3 by C3 convertase C3bBbP	[8] (Optimization)
K_C3_m_C3bBbP	1.80000e-06	M	Cleavage of C3 by C3 convertase C3bBbP	[8] (Optimization)
k_m_C3bBbDAF	1.20000e-03	s^{-1}	Dissociation of DAF complexes on cell	[18]
k_m_C3bBbC3bP	5.70000e-04	s^{-1}	Decay of C3bBbC3bP on cell	[17]
k_pr_surface	1.61000e-12	$M^{-1} s^{-1}$	Production of erythrocytes surface due to physiological turnover	Assumption of steady-state in vivo

k_S	1.34000e-07	s^{-1}	Physiological turnover of erythrocytes	Physiological half-live of 60 days
tau	1.33060e+06	s	Hemolysis scaling coefficient	Derived from PNH patients
k_el_C3	4.61000e-06	s^{-1}	Elimination of C3	Calculated from MW (S4 Appendix, S4 Table)
k_el_C5	4.48000e-06	s^{-1}	Elimination of C5	Calculated from MW (S4 Appendix, S4 Table)
k_el_C6	9.52000e-06	s^{-1}	Elimination of C6	Calculated from MW (S4 Appendix, S4 Table)
k_el_C7	1.15000e-05	s^{-1}	Elimination of C7	Calculated from MW (S4 Appendix, S4 Table)
k_el_C8	5.88000e-06	s^{-1}	Elimination of C8	Calculated from MW (S4 Appendix, S4 Table)
k_el_C9	1.67000e-05	s^{-1}	Elimination of C9	Calculated from MW (S4 Appendix, S4 Table)
k_el_D	6.54000e-05	s^{-1}	Elimination of D	Calculated from MW (S4 Appendix, S4 Table)
k_el_B	1.13000e-05	s^{-1}	Elimination of B	Calculated from MW (S4 Appendix, S4 Table)
k_el_I	1.22000e-05	s^{-1}	Elimination of I	Calculated from MW (S4 Appendix, S4 Table)
k_el_P	2.53000e-05	s^{-1}	Elimination of Properdin	Calculated from MW (S4 Appendix, S4 Table)
k_el_H	5.69000e-06	s^{-1}	Elimination of H	Calculated from MW (S4 Appendix, S4 Table)
k_el_Vn	1.54000e-05	s^{-1}	Elimination of Vn	Calculated from MW (S4 Appendix, S4 Table)
k_el_Cn	1.54000e-05	s^{-1}	Elimination of Cn	Calculated from MW (S4 Appendix, S4 Table)

k_el_iC3b	4.89000e-06	s^{-1}	Elimination of iC3b	Calculated from MW (S4 Appendix, S4 Table)
k_el_Ba	4.65000e-05	s^{-1}	Elimination of Ba	Calculated from MW (S4 Appendix, S4 Table)
k_el_Bb	2.12000e-05	s^{-1}	Elimination of Bb	Calculated from MW (S4 Appendix, S4 Table)
k_el_C3a	1.27545e-04	s^{-1}	Elimination of C3a	[19,20]
k_el_C5a	1.19766e-04	s^{-1}	Elimination of C5a	[21,22]
k_el_C3dg	3.81000e-05	s^{-1}	Elimination of C3dg	[19,23]
k_pr_C3	4.40000e-11	$M\ s^{-1}$	Production of C3	Derived based on in vivo steady-state
k_pr_C5	1.66000e-12	$M\ s^{-1}$	Production of C5	Derived based on in vivo steady-state
k_pr_C6	4.77000e-12	$M\ s^{-1}$	Production of C6	Derived based on in vivo steady-state
k_pr_C7	5.73000e-12	$M\ s^{-1}$	Production of C7	Derived based on in vivo steady-state
k_pr_C8	2.11000e-12	$M\ s^{-1}$	Production of C8	Derived based on in vivo steady-state
k_pr_C9	1.49000e-11	$M\ s^{-1}$	Production of C9	Derived based on in vivo steady-state
k_pr_D	5.33000e-12	$M\ s^{-1}$	Production of D	Derived based on in vivo steady-state
k_pr_B	9.00000e-11	$M\ s^{-1}$	Production of B	Derived based on in vivo steady-state
k_pr_P	1.19000e-11	$M\ s^{-1}$	Production of P	Derived based on in vivo steady-state
k_pr_I	4.91000e-12	$M\ s^{-1}$	Production of I	Derived based on in vivo steady-state
k_pr_H	1.83000e-11	$M\ s^{-1}$	Production of H	Derived based on in vivo steady-state

k_pr_Vn	9.24000e-11	$M\ s^{-1}$	Production of Vn	Derived based on in vivo steady-state
k_pr_Cn	6.62000e-12	$M\ s^{-1}$	Production of Cn	Derived based on in vivo steady-state
k_pr_CR1	1.11000e-15	$M\ s^{-1}$	Production of CR1	Derived based on in vivo steady-state
k_pr_DAF	3.61000e-15	$M\ s^{-1}$	Production of DAF	Derived based on in vivo steady-state
k_pr_CD59	2.81000e-14	$M\ s^{-1}$	Production of CD59	Derived based on in vivo steady-state
kon_ecu	2.31000e-04	$nM^{-1}\ s^{-1}$	kon of eculizumab	Typical mAb on-rate [24,25]
koff_ecu	2.78000e-05	s^{-1}	koff of eculizumab	Derived from kon and KD [26]
k_el_ecu	5.94000e-07	s^{-1}	Elimination of eculizumab	[27]

References

1. Pangburn MK, Schreiber RD, Müller-Eberhard HJ. Formation of the initial C3 convertase of the alternative complement pathway. Acquisition of C3b-like activities by spontaneous hydrolysis of the putative thioester in native C3. J Exp Med. 1981;154: 856–67.
2. Zewde N, Gorham RD, Dorado A, Morikis D. Quantitative Modeling of the Alternative Pathway of the Complement System. PLoS One. 2016;11: e0152337.
3. Bernet J, Mullick J, Panse Y, Parab PB, Sahu A. Kinetic analysis of the interactions between vaccinia virus complement control protein and human complement proteins C3b and C4b. J Virol. 2004;78: 9446–9457.
4. Pangburn MK, Müller-Eberhard HJ. Kinetic and thermodynamic analysis of the control of C3b by the complement regulatory proteins factors H and I. Biochemistry. 1983;22: 178–85.
5. Pangburn MK, Müller-Eberhard HJ. The C3 convertase of the alternative pathway of human complement. Enzymic properties of the bimolecular proteinase. Biochem J. 1986;235: 723–30.
6. Müller-Eberhard HJ, Miescher PA. Complement. Springer-Verlag; 1985.
7. Chen H, Ricklin D, Hammel M, Garcia BL, McWhorter WJ, Sfyroera G, et al. Allosteric inhibition of complement function by a staphylococcal immune evasion protein. Proc Natl Acad Sci. 2010;107: 17621 LP-17626.
8. Korotaevskiy AA, Hanin LG, Khanin MA. Non-linear dynamics of the complement system activation. Math Biosci. 2009;222: 127–43.
9. Muller-Eberhard HJ. The Membrane Attack Complex of Complement. Annu Rev Immunol. 1986;4: 503–528.

10. Cooper NR, Müller-Eberhard HJ. The reaction mechanism of human C5 in immune hemolysis. J Exp Med. 1970;132: 775–793.
11. Li CKN, Levine RP. Rate processes in the final stage of complement hemolysis. Immunochemistry. 1977;14: 421–8.
12. Podack ER, Biesecker G, Kolb WP, Müller-Eberhard HJ. The C5b-6 Complex: Reaction with C7, C8, C9. J Immunol. 1978;121: 484 LP-490.
13. McDonald JF, Nelsestuen GL. Potent Inhibition of Terminal Complement Assembly by Clusterin: Characterization of Its Impact on C9 Polymerization. Biochemistry. 1997;36: 7464–7473.
14. Rawal N, Pangburn MK. C5 convertase of the alternative pathway of complement. Kinetic analysis of the free and surface-bound forms of the enzyme. J Biol Chem. 1998;273: 16828–35.
15. Rawal N, Pangburn M. Formation of high-affinity C5 convertases of the alternative pathway of complement. J Immunol. 2001;166: 2635–42.
16. Pangburn MK, Rawal N. Structure and function of complement C5 convertase enzymes. Biochem Soc Trans. 2002;30: 1006–10.
17. Hourcade DE. The Role of Properdin in the Assembly of the Alternative Pathway C3 Convertases of Complement *. J Biol Chem. 2006;281: 2128–2132.
18. Harris CL, Abbott RJM, Smith RA, Morgan BP, Lea SM. Molecular Dissection of Interactions between Components of the Alternative Pathway of Complement and Decay Accelerating Factor (CD55)*. 2005;280: 2569–2578.
19. Nilsson B, Ekdahl KN. Complement diagnostics: concepts, indications, and practical guidelines. Clin Dev Immunol. 2012;2012: 962702.
20. Norda R, Schött U, Berséus O, Åkerblom O, Nilsson B, Ekdahl KN, et al. Complement activation products in liquid stored plasma and C3a kinetics after transfusion of autologous plasma. Vox Sang. 2012;102: 125–133.
21. Oppermann M, Götze O. Plasma clearance of the human C5a anaphylatoxin by binding to leucocyte C5a receptors. Immunology. 1994;82: 516.
22. Kirschfink M, Mollnes TE. Modern complement analysis. Clin Diagn Lab Immunol. 2003;10: 982–9.
23. Teisner B, Brandslund I, Grunnet N, Hansen LK, Thellesen J, Svehag SE. Acute complement activation during an anaphylactoid reaction to blood transfusion and the disappearance rate of C3c and C3d from the circulation. J Clin Lab Immunol. 1983;12: 63–67.
24. Landry JP, Ke Y, Yu G-L, Zhu XD. Measuring affinity constants of 1450 monoclonal antibodies to peptide targets with a microarray-based label-free assay platform. J Immunol Methods. 2015;417: 86–96.
25. Liu S, Zhang H, Dai J, Hu S, Pino I, Eichinger DJ, et al. Characterization of monoclonal antibody's binding kinetics using oblique-incidence reflectivity difference approach. MAbs. 2015;7: 110–9.
26. Rother RP, Rollins SA, Mojcik CF, Brodsky RA, Bell L. Discovery and development of the complement inhibitor eculizumab for the treatment of paroxysmal nocturnal hemoglobinuria. Nat Biotechnol. 2007;25: 1256–64.
27. Alexion Pharmaceuticals I. Soliris (eculizumab) [Package Insert]. US Food Drug Adm

website https//www.accessdata.fda.gov/drugsatfda_docs/label/2019/125166s431lbl.pdf. 2019; Revised June, 2019. Accessed September, 2019. LP-.
