# Supplementary material for: Modeling the activation of the alternative complement pathway and its effects on hemolysis in health and disease
Source: PLoS Comput Biol. 2020 Oct 2;16(10):e1008139. doi: 10.1371/journal.pcbi.1008139 (PMC7531836; doi:10.1371/journal.pcbi.1008139)
Supplement: S3 Appendix — Derivation of quantitative relationship between MAC formation and hemolysis. (PDF) [file pcbi.1008139.s011.pdf]

Takeda and colleagues [1] investigated the number of hits necessary for MAC-mediated hemolysis through experiments with sheep erythrocytes. In one study the cells were sensitized with preassembled human C5b-7 complexes (EAC1-7 cells), then treated with fixed amounts of C8 and excess of C9 (100 molecules/cell). At low cell concentrations, C8 amounts were sufficient to attain 100% hemolysis. As the number of cells in the incubation increased, C8 became the limiting component for the formation of complete, functional MAC structures. Saturation of the number of lysed cells was observed (Figure in S3 Appendix), indicating that a single MAC is sufficient for hemolysis [1], in accord with the so-called one-hit theory [2].
One limitation of this experimental setup is the unknown amount of C8 in the incubation (described in [1] as 1/3’000 and 1/9’000 dilutions of a C8 preparation). These were estimated as follows. At the cell concentration where saturation begins (marked with an arrow in the Figure in S3 Appendix, 1/3’000 dilution), it was assumed that each cell carries one MAC complex. This is assuming that all C8 molecules bind to the available C5b-7 complexes. i.e. ~$1.35*10^7$ C8 molecules for the 1/9’000 dilution and 3-fold as many for the 1/3'000 dilution. Therefore, the number of MAC complexes could not increase beyond 1.35*107 and 4.05*107 for the two dilutions. The percentage of lysed cells (calculated as $\frac{Number\ of\ lysed\ cells}{Number\ of\ EAC1\text{–}7\ cells} \cdot 100\%$)and the number of MAC complexes per cells ($\frac{Number\ of\ MAC}{Number\ of\ EAC1\text{–}7\ cells}$) were used to derive the MAC-hemolysis relationship presented in Fig 3.

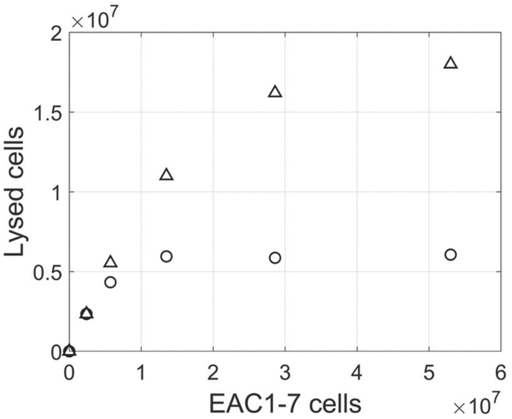

Figure. Hemolysis as a function of cell number and C8 concentration in incubation. Sheep EAC1-7 erythrocytes were treated with 1/3'000 (circles) or 1/9’000 (triangles) dilutions of a C8 preparation and excess of C9. Saturation of lysis, corresponding to one MAC/cell, was assumed to begin at the marked cell concentration (arrow). The data was digitized from Fig 2 in [1].

References
1. Takeda J, Kozono H, Takata Y, Hong K, Kinoshita T, Sayama K, et al. Number of hits necessary for complement-mediated hemolysis. Microbiol Immunol. 1986;30: 461–8.
2. Mayer MM. Development of the one-hit theory of immune hemolysis. Immunochem Approaches to Probl Microbiol. 1961; 268–279.
