# Supplementary material for: Modeling the activation of the alternative complement pathway and its effects on hemolysis in health and disease
Source: PLoS Comput Biol. 2020 Oct 2;16(10):e1008139. doi: 10.1371/journal.pcbi.1008139 (PMC7531836; doi:10.1371/journal.pcbi.1008139)
Supplement: S4 Appendix — Quantitative relationship between molecular size and half-life. (PDF) [file pcbi.1008139.s012.pdf]

Schmidt and Wittrup [1] previously reported data for a set of macromolecules on their molecular size and systemic plasma clearance in the mouse. Based on their work, we described the relationship between the hydrodynamic radius, R_i, and elimination rate constant, $k^m_{el,i}$, for molecule i with the following formula:

$$k^m_{el,i} = \frac{k_{max}}{\delta^{\left(\frac{R_i{}^\gamma}{R_i{}^\gamma + k_{50}{}^\gamma}\right)}} \quad \text{(Eq. S1)}$$

under the assumption of first-order elimination kinetics. We fitted the log-transformed data of supplementary table S4 in [1] and estimated the following parameter values: k_{max} =5.37 hr^{-1}, δ= 165.96, k_{50} = 3.87 nm, and γ = 3.39 (Figure in S4 Appendix). IgG data were excluded from the analysis because of the prolongation in half-life mediated by neonatal Fc receptor recycling.

In order to use Eq. S1 for calculating the elimination rate constant of complement proteins, their hydrodynamic radius was estimated from the molecular weight, MW_i, using Eq. S2 [1]:

$$R_i = 0.912 \cdot MW_i{}^{0.333} \quad \text{(Eq. S2)}$$

The rate constant of plasma elimination in human was scaled allometrically from the mouse value using Eq. S3, assuming a value of -0.25 for the scaling exponent:

$$k^h_{el,i} = k^m_{el,i} \cdot \left(\frac{BW^h}{BW^m}\right)^{-0.25} \quad \text{(Eq. S3)}$$

where BW is the body weight, assumed to be 70 and 0.02 kg for human and mouse, respectively. With Eq. S1-S3, the plasma elimination half-life of complement proteins was calculated for use in the simulations of human complement activity in vivo (S4 Table).

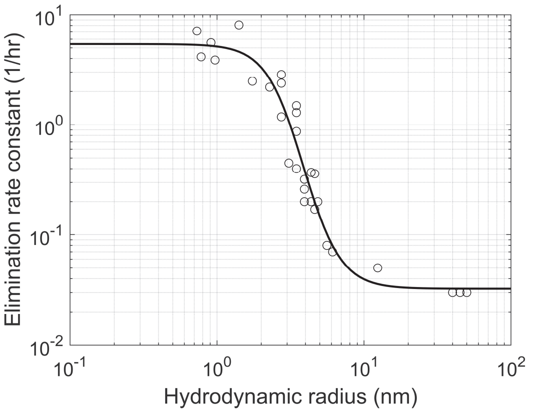

Figure. Plasma elimination of macromolecules in mice as a function of molecular radius. Elimination rate constant values and estimated hydrodynamic radius of macromolecules (circles) were obtained from supplementary table S4 in [1]. The data were fitted with the sigmoid function of Eq. S1 (line).

Reference

1. Schmidt MM, Wittrup KD. A modeling analysis of the effects of molecular size and binding affinity on tumor targeting. Mol Cancer Ther. 2009;8: 2861–71.
